# Asymmetric Active Center Triggers Side‐On Photo‐Fenton‐Like Reaction Through Polar Charge Transfer and Self‐Adapting Sulfur Vacancies

**DOI:** 10.1002/advs.202517517

**Published:** 2026-02-10

**Authors:** Jiaqi Hu, Xiaoyuan Zhang, Yufei Gao, Jianyi Liu, Xu He, Yu Liu, Jinfeng Lu, Jun Ma

**Affiliations:** ^1^ Tianjin Key Laboratory of Environmental Technology For Complex Trans‐Media Pollution Key Laboratory of Pollution Processes and Environmental Criteria of Ministry of Education College of Environmental Science and Engineering of Nankai University Tianjin China; ^2^ State Key Laboratory of Urban Water Resource and EnvironmentHarbin Institute of Technology Harbin China

**Keywords:** asymmetric sites, photo‐fenton‐like, polar charge transfer, self‐adapting, side‐on PMS activation

## Abstract

The Fenton‐like process driven by visible‐light (photo‐Fenton‐like) holds immense promise for abating emerging contaminants through offering efficient charge transfer in peroxymonosulfate (PMS) activation. However, the catalytic interface charge transfer limitations, weak adsorption interactions, and high dissociation energy of the short O─O bond hinders efficient PMS activation in these systems. Herein, asymmetric active centers were constructed in a Cu‐doped Zn_3_In_2_S_6_ photocatalyst (Cu–ZIS_V_) to accelerate PMS activation kinetics via modulating the polar charge transfer and activation pathway of PMS. The resulting asymmetric Cu–S_V_–Zn sites resulted in an increase in the first‐order kinetic constant of Cu–ZIS_V_ by 4.87 times (0.1674 min^−^^1^) through polar charge transfer, outperforming the previously reported sulfur vacancy‐based photocatalysts. Self‐adapting S vacancies triggered by Cu‐doping regulated the traditional activation of PMS from end‐on‐type to side‐on‐type. The synergistic effect of polar charge transfer and the side‐on surface‐bound PMS complex (side‐on PMS*) facilitated the stretching of O─O bonds (1.362–1.493 Å) in PMS and lowered the free energy barrier by 85% (1.176–0.175 eV), significantly promoting the generation of reactive oxygen species (ROS). This study offers new insights for improving the efficiency of side‐on photo‐Fenton‐like reactions via self‐adapting modulation, providing a promising strategy for efficient photo‐Fenton‐like processes in practical water treatment.

## Graphical abstract

The Cu‐Zn_3_In_2_S_6_ photocatalyst (Cu‐ZIS_V_) with asymmetric Cu‐SV‐Zn sites was designed and synthesized to boost peroxymonosulfate (PMS) activation kinetics. The side‐on photo‐Fenton‐like reaction by the asymmetric Cu‐S_V_‐Zn site accelerated PMS activation and pollutant degradation, and the first‐order kinetic constant of Cu‐ZIS_V_ increased by 4.87 times.

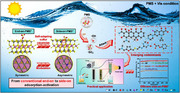

## 1 Introduction

Rapid urbanization and industrialization have resulted in the widespread release of emerging contaminants (ECs) into water ecosystems [1]. Owing to persistence and bioaccumulation, these ECs pose long‐term and unpredictable risks to both ecosystems and human health [2], emphasizing the urgent need for efficient and sustainable treatment solutions. Among advanced oxidation processes (AOPs), peroxymonosulfate (PMS)‐based systems have been shown to facilitate the generation of reactive oxygen species (ROS), notably singlet oxygen (^1^O_2_, E_0_ = 1.88 V_NHE_), hydroxyl radicals (•OH, E_0_ = 1.80–2.70 V_NHE_), and sulfate radicals (•SO_4_^−^, E_0_ = 2.50–3.10 V_NHE_), which are crucial for effective pollutant degradation and removal of ECs [3, 4]. While various metal oxides or metal hydroxides can activate PMS, they are generally subject to limitations [5, 6]. (i) The low transfer efficiency of electrons and insufficient cyclic dynamics of metal valence changes at the active sites lead to catalyst deactivation and increase the application cost. (ii) The oxidation or precipitation of transition metal ions leads to low catalyst utilization. (iii) Approximately 30–50% of PMS is activated to ^1^O_2_ (weak oxidizing capacity), resulting in low‐selectivity generation of •OH/•SO_4_^−^ and consequently decreasing the mineralization efficiency of ECs. Photocatalytic activation of PMS using low‐cost semiconductors under visible‐light (Vis) irradiation offers significant environmental and economic advantages. Since photoexcited PMS primarily concentrates the electron cloud in O─O bonds (Figure S1), semiconductors with appropriate band structures can generate photogenerated electrons to facilitate photo‐Fenton‐like systems (breaking O─O bonds in PMS molecules) [7]. These photogenerated electrons regenerate active sites, reduce PMS consumption, and increase ROS production, thus enhancing the degradation and mineralization of ECs. Nevertheless, the overall activation efficiency is often limited by ineffective interfacial charge transfer and weak adsorption molecular polarization, despite the potential of visible‐light‐induced electrons to enhance reaction kinetics [8−10].

The initial and critical step in PMS activation is the formation of a surface‐bound PMS complex (PMS*) via the adsorption‐activation of PMS over catalysts [11−13], with the conformation between PMS and the catalyst influencing both O─O bond cleavage and the resulting ROS pathway. Conventional end‐on‐type adsorption conformations with PMS hinder the cleavage of the O─O bond, negatively influencing the charge transfer efficiency of the active site and limiting activation efficiency [14, 15]. In contrast, a side‐on adsorption mode, where both oxygen atoms in PMS interact with the site, can elongate the O─O bond and lower the dissociation energy, thus enhancing ROS production [16]. Recently, the construction of atomic‐level defects has opened ideas for specific interface contact adsorption [17, 18]. Defective sites with localized electrons and unsaturated coordination atoms can serve as effective anchor sites for PMS [19]. At these sites, both oxygen atoms in the O─O bond of PMS can be simultaneously adsorbed by symmetric cations surrounded by defects, and the electron‐rich environments provide sufficient activation energy. However, owing to the strong binding of electrons at highly symmetric sites, a uniform electron distribution around these cations may impede efficient charge transfer between PMS and the catalysts. This intrinsic limitation underscores the need for new strategies to break the charge uniformity and enhance electron mobility at the catalytic interface.

Asymmetric vacancies, characterized by uneven coordination environments, can effectively disrupt this uniform electron distribution and introduce locally polarized electronic structures [20]. In contrast to symmetric vacancies (M_1_‐V‐M_1_), asymmetric vacancies (M_1_‐V‐M_2_) exhibit locally polarized electron features, enabling the modulation of active sites through different coordination numbers and dangling bonds [21]. Notably, the bridging adsorbed species display markedly different charge distributions as a result of the electron distributions and distinct valence states of the M_1_ and M_2_ atoms, which in turn tune polar charge transfer and break chemical bonds [22, 23]. This distinguishing charge asymmetry property improved the adsorption and polarization of PMS molecules, making efficient PMS‐based AOPs more achievable [24]. Despite their promise, the catalytic mechanism of asymmetric vacancies—especially in sulfur‐based systems—remains controversial. In the M_1_‐V‐M_2_ configuration, the vacancy serves as a vital mediator and electron trap, significantly influencing the spatial distribution and transfer of polar charges between M_1_ and M_2_ atoms [21]. Furthermore, S vacancies are known to regulate the electronic structure of adjacent metal atoms and reduce energy barriers for the decomposition of reaction intermediates, thus enhancing the overall photocatalytic performance of the system [25]. These findings collectively support the concept of using side‐on PMS activation pathways enabled by asymmetric coordination environments. However, few studies have focused on the precise construction mechanism of asymmetric adaptive S vacancies and their stability evolution, which is crucial for the rational design of S vacancy‐based photocatalysts with high activity and stability. The details of the atomic‐level interactions and side‐on modulation mechanisms of self‐adapting active centers in photo‐Fenton‐like processes remain poorly understood.

Zn_3_In_2_S_6_ has emerged as a promising layered transition metal chalcogenide for photocatalytic PMS activation because of its unique layered stable 2D structure and suitable band configuration [26, 27]. Doping copper (Cu) atoms, with an atomic number adjacent to zinc (Zn), maximizes the visible‐light absorption and structural stability without changing the 2D layered structure of Zn_3_In_2_S_6_. More importantly, the substitution of Zn atoms by Cu atoms can cause Jahn–Teller distortion to form self‐adapting S vacancies [28]. The relatively short and weak Cu─S bonds lead to Cu─S shrinkage, thus constricting the ZnS layer and broadening the interlayer spacing, which is rarely observed in metal oxides or metal hydroxides. In this study, we designed and synthesized Cu–Zn_3_In_2_S_6_ photocatalysts featuring asymmetric Cu–S_V_–Zn sites to realize a side‐on photo‐Fenton‐like oxidation pathway for degrading ECs. Tetracycline (TC) was selected as a representative EC to evaluate its catalytic performance and practical application potential [10, 29]. Through advanced characterization, the formation of asymmetric active sites induced by Cu‐doping and self‐adapting S vacancies, as well as the resultant polar charge transfer, was systematically elucidated. More importantly, this work investigated the correlation between asymmetric active sites and PMS activation kinetics, first demonstrating the modulation mechanism of asymmetric Cu–S_V_–Zn sites on the side‐on photo‐Fenton‐like oxidation pathway. Finally, the anti‐interference ability and practical application potential of the side‐on photo‐Fenton‐like oxidation pathway in wastewater were evaluated. This work highlights the potential of precisely engineered vacancies to regulate the efficiency of side‐on photo‐Fenton‐like oxidation pathways, offering crucial insights and guiding principles for the rational design of photo‐Fenton‐like processes for water purification.

## 2 Results and Discussion

### 2.1 Characterization of Catalysts with Self‐Adapting Sulfur Vacancies

**FIGURE 1:**
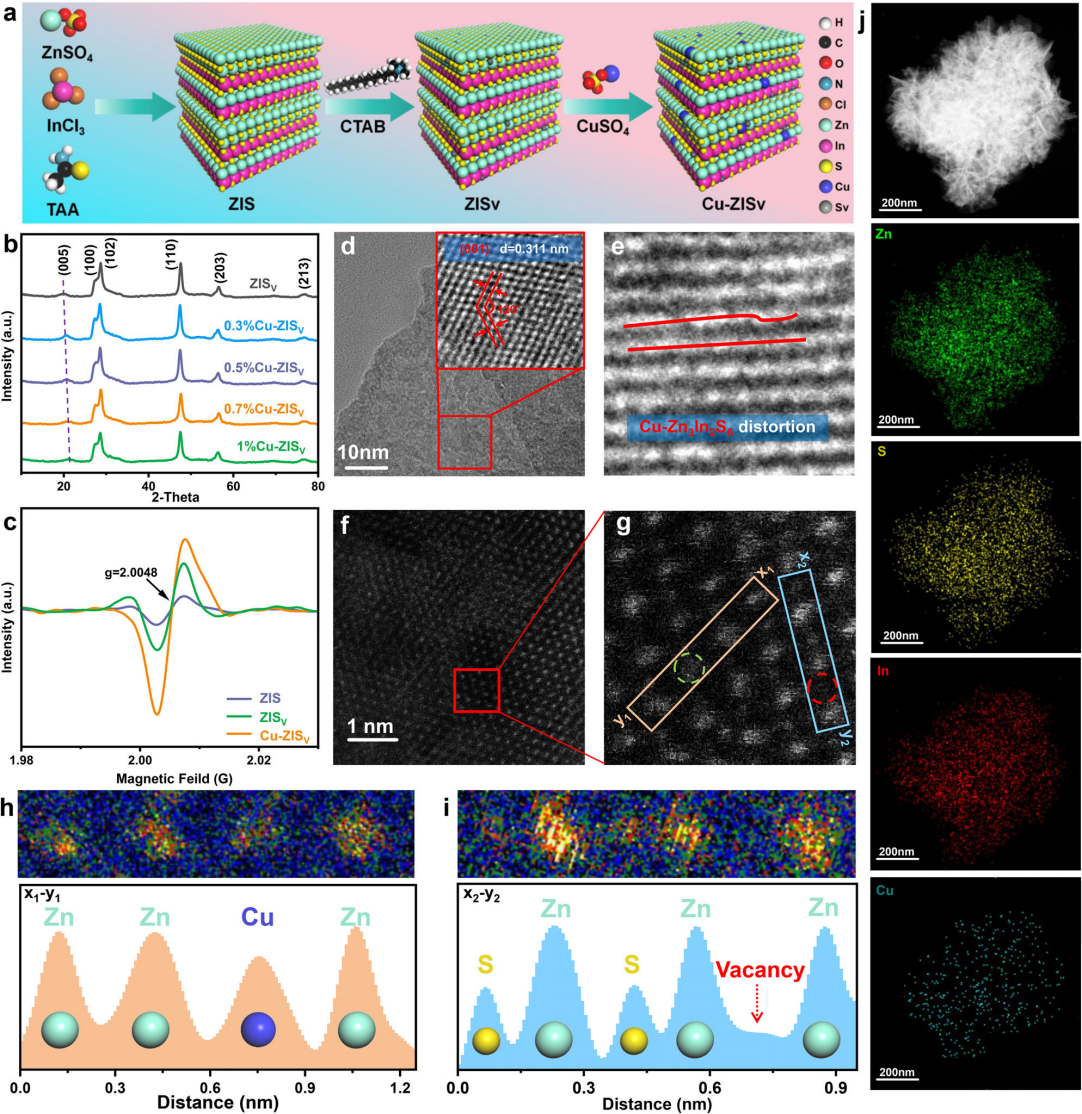
(a) Schematic of the preparation of Cu–ZIS_V_. (b) XRD patterns and (c) EPR spectra of the samples. TEM images of (d) ZIS_V_ and (e) Cu–ZIS_V_. (f,g) Atomic resolution HAADF–STEM images of Cu–ZIS_V_. (h,i) Line scan measured along the x–y rectangular regions. (j) EDS‐mapping images of Cu–ZIS_V._

To investigate the underlying factors of the difference in catalytic activity and stability among the various synthesized catalysts, a series of structural and compositional characterizations were carried out. The synthesis of Cu–ZIS_V_ via a one‐step hydrothermal method is schematically illustrated in Figure 1a. The Cu–ZIS_V_ composite with a Cu loading of 0.7% exhibited the highest photocatalytic activity among all the tested samples and was therefore chosen as the model photocatalyst for further study (Figures S2–S4).

The X‐ray diffraction (XRD) pattern shows that the diffraction peaks of the photocatalyst are primarily associated with a hexagonal phase of Zn_3_In_2_S_6_ (Figure 1b, JCPDS No. 24–1453) [30], with the shift of the (005) reflection to a higher angle affirming poor crystalline symmetry and lattice shrinkage [26]. Compared with that of ZIS, the electronic paramagnetic resonance (EPR) spectrum of Cu–ZIS_V_ exhibits a significantly increased signal intensity, implying that Cu doping induced an increase in the number of self‐adapting S vacancies and reinforced the catalyst asymmetry (Figure 1c) [28, 31]. Transmission electron microscope (TEM) images confirm an interplanar distance of 0.311 nm, corresponding to the (001) crystal plane of ZIS_V_ (Figure 1d; Figure S5). Additionally, the observed interaxial angles of 120° are highly consistent with those of the expected hexagonal crystal structure. In addition, compared with the pristine (001) phase, a slight lattice distortion in Cu–ZIS_V_ can be distinguished upon the introduction of Cu atoms (Figure 1e; Figure S6). High‐angle annular dark‐field scanning transmission electron microscopy (HAADF‐STEM, Figure 1f,g) reveals an atomic column brightness consistent with the atomic number sequence Zn > Cu [32], allowing Cu atoms to be distinguished as lower‐intensity spots (green circles). Furthermore, the line intensity profile analysis in Figure 1h, further confirms that some Zn atoms are substituted by Cu atoms, accompanied by the formation of S vacancies. The element mapping images unambiguously illustrate that the elements Zn, In, S, and Cu were dispersed homogeneously in the Cu–ZIS_V_ (Figure 1j). The formation of asymmetric structures in Cu–ZIS_V_ could inhibit charge transfer between layers and promote PMS activation.

**FIGURE 2:**
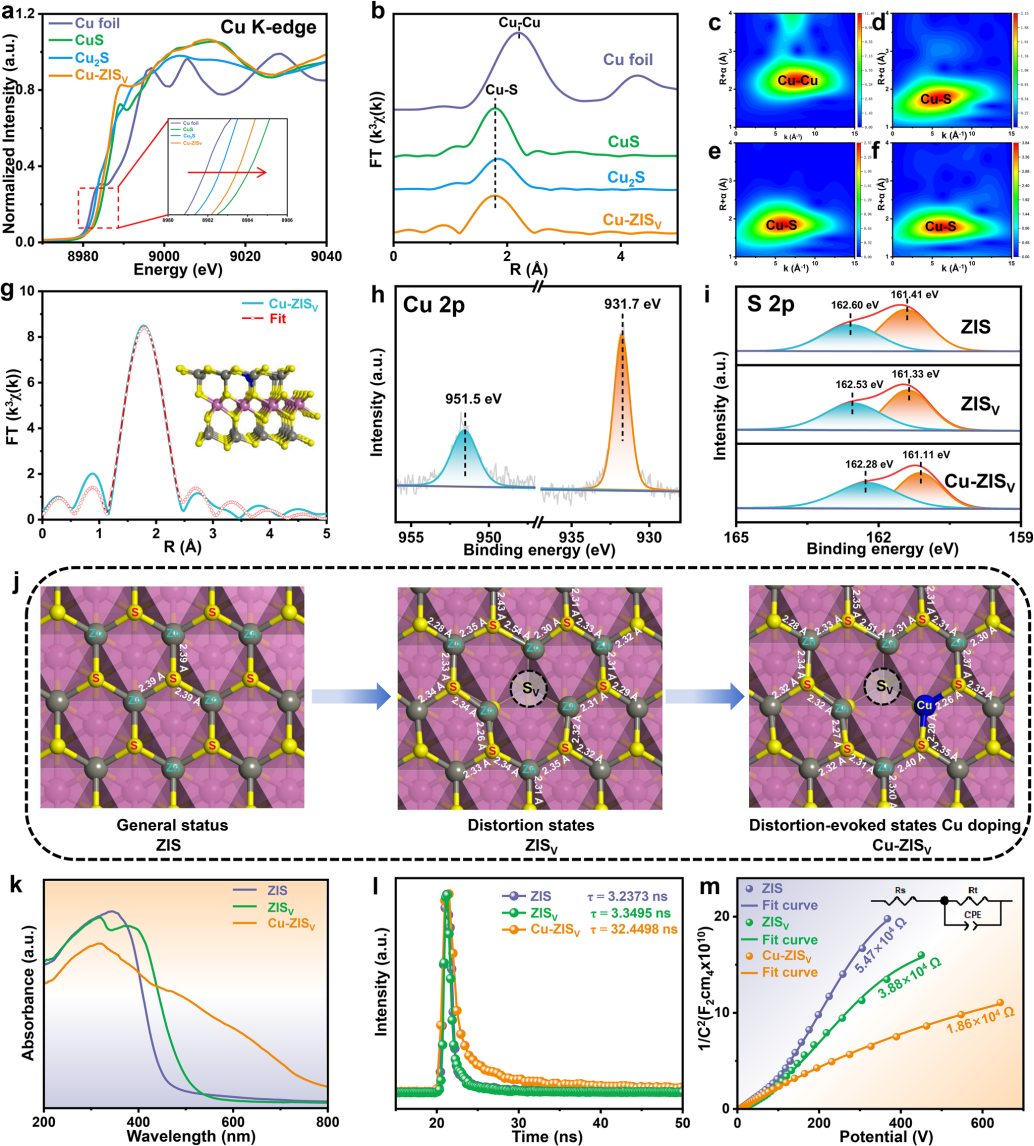
(a) XANES spectra of Cu foil, CuS, Cu_2_S, and Cu–ZIS_V_. (b) Fourier transform spectra of k^3^‐weighted Cu K‐edge EXAFS in Cu foil, CuS, Cu_2_S, and Cu–ZIS_V_. Cu K‐edge wavelet transform–EXAFS contour plots for (c) Cu foil, (d) CuS, (e) Cu_2_S, and (f) Cu–ZIS_V_. (g) The corresponding EXAFS fitting results of Cu–ZIS_V_ in R space. XPS spectra for the (h) Cu 2p and (i) S 2p regions. (j) Schematic of the local structure transformation and corresponding bond lengths of Cu–ZIS_V_ to form distortion and Cu‐doping. (k) UV–vis diffuse reflectance spectra, (l) time‐resolved fluorescence decay spectra, and (m) EIS spectra of ZIS, ZIS_V_, and Cu–ZIS_V._

The electrical structure and chemical environment of Cu doped into Cu–ZIS_V_ were thoroughly investigated by XAFS measurements. The absorption edge of Cu in Cu–ZIS_V_ (≈8982 eV) is located between CuS and Cu_2_S, exhibiting a chemical state ranging from +1 to +2, as shown in the Cu K edge X‐ray absorption near‐edge structures (XANES) (Figure 2a). This result may be caused by the imbalance in electrons, indicating the presence of a defect state and polarity charge distribution surrounding the Cu site [33, 34]. In contrast to the Cu─Cu bond in Cu foil (≈2.2 Å), Cu–ZIS_V_ reveals a distinct signal peak at ≈1.8 Å corresponding to the Cu−S bond, suggesting the absence of Cu─Cu bonds and excellent dispersion of Cu atoms (Figure 2b). The wavelet transforms analysis of Cu–ZIS_V_ revealed that the Cu−S bond signal was present at approximately 5.6 Å (Figure 2c–f), implying that Cu atoms were integrated into ZIS_V_ by replacing Zn atoms to form the Cu−S bond. This conclusion can also be verified by ICP–MS (Table S1). Additionally, extended X‐ray absorption fine structure (EXAFS) fitting analysis revealed a coordination number (CN) of 3.1 for Cu atoms in Cu–ZIS_V_, which is significantly lower than those of CuS (3.7) and Cu_2_S (3.8) (Figure 2g; Figure S7 and Table S2). This phenomenon is primarily associated with the direct connection between Cu atoms and S vacancies, leading to the absence of a coordinated S atom by the Cu atom and significantly enhancing electron transfer for PMS activation.

XPS was performed to determine the chemical states of the samples (Figure 2h; Figures S8 and S9). The two major peaks of Cu–ZIS_V_ at 931.7 and 951.5 eV are attributed to Cu 3d_5/2_ and Cu 3d_3/2_, respectively, in the Cu 3d region, confirming the presence of Cu species mainly in the form of Cu^2+^ [35, 36]. The S 2p XPS spectrum moves toward lower binding energy with increasing S vacancies, which redistribute additional electrons to nearby cation sites (Figure 2i; Figure S10 and Tables S3 and S4) [37, 38]. DFT calculations of the distortion states and S vacancy structures evoked by Cu doping revealed that the lengths of the Cu─S bond decreased from 2.39 to 2.20 and 2.26 Å, respectively (Figure 2j). The distortion state of the metal−S bond (Zn−S/Cu−S) reduced the CN of Zn/Cu atoms and increased the number of S vacancies through local structural self‐adapting, which subsequently induced the formation of asymmetric Cu–S_V_–Zn sites. The substitution of Zn^2+^ (radius of 0.60 Å) by the smaller Cu^2+^ (radius of 0.57 Å) induced localized compressive strain within the lattice, which generated both compressive and tensile stresses, accompanied by the formation of charge defects. [28] In this state, the system actively expelled S^2−^ to reduce the total energy and relieve the in‐plane compressive stress. Under hydrothermal conditions, the dissolution–readsorption equilibrium of S^2−^ remained dynamic. The localized tensile stress area surrounding Cu^2^^+^ became the preferred dissolution site for S^2−^. Once S vacancies formed, they were relocked by surrounding metal–S bonds, achieving “self‐adaptive” stabilization and producing self‐adaptive S vacancies. UV–vis diffuse reflection spectroscopy revealed that the substitution between Zn atoms and Cu atoms caused by Cu doping improved the visible‐light absorption ability of the photocatalyst, resulting in full visible‐spectrum absorption (380–760 nm) of Cu–ZISv (Figure 2k; Figures S11 and S12). The data in Figure 2l,m suggest that Cu–ZIS_V_ has the highest separation and migration efficiency of photogenerated electron–hole pairs [39, 40, 41]. Collectively, these results indicate that the asymmetric Cu–S_V_–Zn sites induced by self‐adapting S vacancies allow faster and more efficient polar electron transfer, which is crucial for the activation of PMS (Figure 2m; Figure S13 and Table S5).

### 2.2 Catalytic properties of the Cu–ZIS_V_ + PMS + Vis System

**FIGURE 3:**
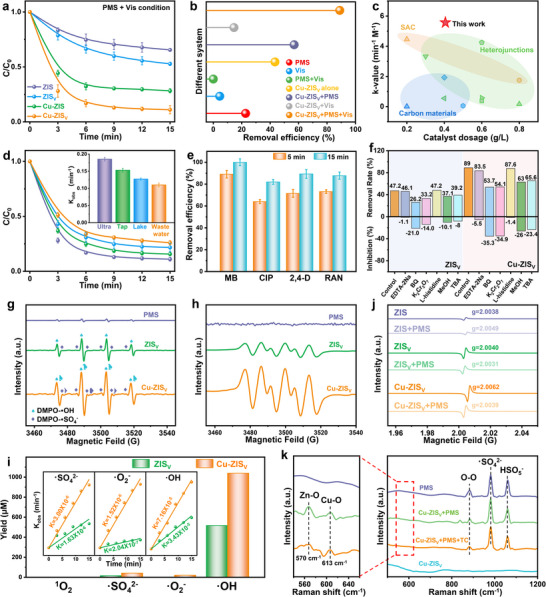
(a) Degradation efficiency of TC by ZIS, ZIS_V_, Cu–ZIS, and Cu–ZIS_V_ under PMS + Vis conditions. (b) Degradation efficiency of TC under different combinations of Cu–ZIS_V_, PMS, and visible‐light. (c) Comparison of various advanced catalytic materials. (d) Degradation efficiency of TC by the Cu–ZIS_V_ + PMS + Vis system in various water bodies. (e) Multiple contaminants degradation by the Cu–ZIS_V_ + PMS + Vis system. (f) Degradation efficiency of TC by ZIS_V_ and Cu–ZIS_V_ with different scavengers. (g) EPR spectra of •OH and •SO_4_^−^. (h) EPR spectra of •O_2_^−^. (i) The quantities of ROS produced by ZIS_V_ and Cu–ZIS_V_. (j) In situ ESR spectra of ZIS, ZIS_V_, and Cu–ZIS_V_. (k) In situ Raman spectra of PMS, Cu–ZIS_V_ + PMS, Cu–ZIS_V_ + PMS + TC, and Cu–ZIS_V_.

To evaluate the effect of self‐adapting modulation of polar charge transfer from asymmetric Cu–S_V_–Zn sites on PMS activation, the photo‐Fenton‐like catalytic performance of Cu–ZIS_V_ for TC removal was systematically investigated. Cu–ZIS_V_ achieved the highest photodegradation efficiency among the catalysts, degrading 92.7% of TC within 15 min after achieving adsorption–desorption equilibrium (Figure 3a; Figures S14 and S15). The rate constant (*k_obs_*) of Cu–ZIS_V_ (0.1674 min^−1^) was 4.87 times that of ZIS_V_ (0.0344 min^−1^), demonstrating that Cu species serve as the major catalytic active center for PMS activation. This enhancement highlights the critical role of the Cu coordination environment in tuning PMS activation. Furthermore, the degradation efficiencies of TC in the PMS + Vis, Cu–ZIS_V_ + Vis, and Cu–ZIS_V_ + PMS + Vis systems were 23%, 15%, and 93%, respectively, indicating that photogenerated electrons could be enriched in Cu species to greatly increase PMS activation for EC removal (Figure 3b; Figures S16 and S17). The improved TC removal performance may be attributed to two factors: (i) the increase in self‐adapting S vacancies on the ZIS_V_ substrate after doping with Cu atoms (Figure 1c) and (ii) the formation of asymmetric Cu–S_V_–Zn sites (Figure 2j), resulting in a rearrangement of the charge polarization as well as an optimized adsorption–activation conformation of the PMS.

The calculations of the modified kinetic rate constant (*k* value) for the various catalytic systems employed in TC degradation are summarized in Figure 3c and Tables S6 and S7. Notably, the *k* value of Cu–ZIS_V_ reaches an impressive value of 5.57 min^−1^⋅M^−1^, reflecting its superior catalytic activity. In real water samples (secondary sewage from Tianjin, China), the Cu–ZIS_V_ + PMS + Vis system also demonstrated strong resistance and cycling stability to interference from environmental ions, with the TC oxidation efficiency reaching 76% (Figure 3d; Figures S18 and S19). The residual organic matter (473 mg L^−1^ of TOC) and chloride (376 mg L^−1^ of Cl^−^) in wastewater were the main reasons for the decrease in TC degradation efficiency (Table S8): (i) the residual organic matter competed with TC for the •OH/•SO_4_^−^ generated by PMS activation, and (ii) Cl^−^ converted the •OH/•SO_4_^−^ into less reactive secondary radicals or ionic intermediates, thus reducing the effective concentration and oxidative capacity of free radicals in the photo‐Fenton‐like system. As shown in Figure 3e and Figure S20, ciprofloxacin (CIP), methylene blue (MB), 2,4‐dichlorophenoxyacetic acid (2,4‐D), and ranitidine (RAN) were degraded by more than 80% within 15 min. This result demonstrated the wide applicability of the Cu–ZIS_V_ + PMS + Vis system for the degradation of various emerging contaminants. Furthermore, Cu–ZIS_V_ can effectively mineralize 78% of TC into H_2_O and CO_2_ (Figures S21 and S22 and Table S9). The biological toxicity of TC and its intermediates during the degradation process was assessed by the Toxicity Estimation Software Tool (T.E.S.T, version 5.1.1, USEPA). As presented in Table S10, Cu–ZIS_V_ significantly reduced the biological toxicity of TC and its intermediates, indicating the potential of Cu–ZIS_V_ as a promising solution for wastewater treatment. These results confirmed that Cu–ZIS_V_ possessed superior catalytic performance, high recyclability, and strong stability and is promising for application in water treatment.

### 2.3 Mechanism of side‐On Photo‐Fenton‐Like Degradation

#### 2.3.1 Identification of Active Species

To identify the contributions of different active species in the Cu–ZIS_V_ + PMS + Vis system, various quenching agents were employed. As shown in Figure 3f, the inhibitory effects of methanol (MeOH), EDTA‐2Na, L‐histidine, tert‐butanol (TBA), benzoquinone (BQ), and K_2_Cr_2_O_7_ on the degradation of TC provided evidence for the individual role and contributions of •SO_4_^−^, h^+^, ^1^O_2_, •OH, •O_2_^−^ and e^−^, respectively. The addition of BQ and K_2_Cr_2_O_7_ in the Cu–ZIS_V_ + PMS + Vis system led to significant decreases in the oxidation efficiency by 35.3 and 34.9%, respectively, revealing that •O_2_^−^ and e^−^ were crucial for TC degradation in the Cu–ZIS_V_ + PMS + Vis system. Furthermore, MeOH could simultaneously trap both •OH and •SO_4_^−^, whereas TBA trapped mainly •OH, with negligible capture of •SO_4_^−^ [42]. The difference in the inhibition of TC degradation by MeOH and TBA reflected the contribution of •SO_4_^−^. Both MeOH and TBA suppressed the TC degradation efficiency, suggesting that TC degradation is facilitated by the generation of •OH and •SO_4_^−^ from PMS activation. EDTA‐2Na and L‐histidine had negligible effects (5.5% and 1.4%, respectively) on the Cu–ZIS_V_ + PMS + Vis system, which could be attributed to the depletion of PMS by EDTA‐2Na/L‐histidine and competition with organic matter for the adsorption sites on the catalyst. No ^1^O_2_ signal peak was detected by EPR, indicating the absence of ^1^O_2_ in the system using 2,2,6,6‐tetramethyl‐4‐piperidinyl (TEMP) as the ^1^O_2_ capture agent (Figures S23 and S24) [21, 22]. The EPR spectrum in Figure 3g shows DMPO‐•SO_4_^−^ and DMPO‐•OH signals, demonstrating the production of •SO_4_^−^ and •OH species as well as PMS activation. As shown in Figure 3h and Figure S25, there was a strong signal peak of •O_2_^−^ in Cu–ZIS_V_ but not in the single PMS in the EPR spectrum [43], indicating that •O_2_^−^ originated from the activation of molecular oxygen rather than PMS. In general, the EPR results agreed with the results of the quenching experiments.

Quantifying the steady‐state concentration of active species was essential for elucidating the photo‐Fenton‐like process. Although the EPR signal intensity provided relative rates of ROS generation, it could not be directly applied for quantitative analysis. Quantitative probe experiments with furfuryl alcohol (FFA), benzoic acid (BA), nitrobenzene (NB), and nitro‐blue chloride (NBT) were performed to quantify ^1^O_2_, •SO_4_^−^, •OH, and •O_2_^−^ (Figure 3i). In the ZIS_V_ + PMS + Vis system, the steady‐state concentrations of •SO_4_^−^, •OH, and •O_2_^−^ reached 22.41, 517.12, and 2.57 µM, respectively, within 15 mins. Notably, the production amounts of •SO_4_^−^, •OH, and •O_2_^−^ increased significantly to 41.35, 1040.09, and 20.95 µM, respectively, for the Cu–ZIS_V_ + PMS + Vis system. The improved •O_2_^−^ concentration likely originated from the formation of self‐adapting S vacancies, which facilitated effective oxygen adsorption for molecular oxygen activation. Moreover, compared with ZIS_V_, Cu–ZIS_V_ resulted in higher generation rates of •SO_4_^−^, •OH and •O_2_^−^, with values of 3.00 × 10^−6^, 7.16 × 10^−5^ and 1.52 × 10^−6^ mol L^−1^ min^−1^, respectively, which were 1.85 (1.63 × 10^−6^ mol L^−1^ min^−1^), 2.08 (3.43 × 10^−5^ mol L^−1^ min^−1^) and 7.43 times (2.04 × 10^−7^ mol L^−1^ min^−1^) greater, respectively. The contribution rates of •OH, •SO_4_^−^, and •O_2_^−^ to tetracycline degradation in the Cu–ZIS_V_ +PMS +Vis system were calculated to be 82.4, 16.1, and 1.5%, respectively (Table S11). Owing to the steady‐state concentration and contribution rate of •OH far exceeding those of the other free radicals, the •OH generated from highly efficient PMS activation by photogenerated electrons was the main active species in this photo‐Fenton‐like system, further confirming the contribution of the polar charge transfer of asymmetric active centers to PMS activation.

#### 2.3.2 Side‐On Activation Modulation via Self‐Adapting S Vacancies

To investigate the adsorption–activation mechanism of this system, ESR spectra of the catalysts and catalysts/PMS in pure water were recorded. As shown in Figure 3j, ZIS exhibited a weak signal of unpaired electrons (g = 2.0038) [44, 45]. When PMS was added, the g‐value increased to 2.0049, which was attributed to interfacial charge transfer from ZIS to PMS. Owing to the accumulation of unpaired electrons at S vacancies, ZIS_V_ displayed a stronger signal with a g‐value of 2.0040 [46]. After the introduction of PMS, the signal shifted to a lower magnetic field (g = 2.0031), indicating that PMS depleted the unpaired electrons at S vacancies. With respect to Cu–ZIS_V_, the signal strongly shifted upon the addition of PMS, with the g‐value decreasing from 2.0062 to 2.0039, suggesting that Cu single atoms enhanced the catalytic activity of S vacancies and accelerated PMS activation. However, the shift in the g‐value was closely related to the orbital coupling between O 2p in PMS and Zn/Cu 3d in the catalysts and was significantly correlated with the presence of S vacancies [47]. This demonstrated that S vacancies could create a spatially favorable environment for the simultaneous adsorption of dual oxygen atoms of O─O bonds in PMS, thus regulating the adsorption–activation configuration of PMS. Furthermore, the XPS spectra revealed that the Zn 2p peaks of ZIS and ZIS_V_ shifted to higher binding energy by 0.15 and 0.38 eV, respectively, whereas the Cu 2p and Zn 2p peaks of Cu–ZIS_V_ shifted to increased binding energy by 0.52–0.23 eV, respectively (Figures S26 and S27). These findings indicated that PMS received electrons from ZIS, ZIS_V_, and Cu–ZIS_V_, thus excluding the influence of the electron transfer pathway on the shift direction of the g‐value, which in turn confirmed that S vacancies modulated the PMS adsorption–activation configuration.

In situ Raman spectroscopy was utilized to further investigate the interactions between the Cu–S_V_‐Zn sites and PMS (Figure 3k). The signal peaks at 980 and 1060 cm^−1^ correspond to the symmetric stretching vibrations of the S═O bond in SO_4_^2−^ and SO_3_^−^ in HSO_5_^−^, respectively [48]. Interestingly, a new characteristic peak appeared at 835 cm^−1^ in the spectrum of Cu–ZIS_V_ with the addition of PMS. This peak is attributed mainly to the creation of metal–PMS*, a peroxo‐species bond that forms with the surface metal sites. In addition, characteristic peaks corresponding to Zn─O and Cu─O were observed at 570 and 613 cm^−1^, respectively, for Cu–ZIS_V_ + PMS, indicating that the O─O bond of PMS formed side‐on adsorption at the Cu–S_V_–Zn site, which likely further increased the polarity charge distribution of the asymmetric active center [49]. Interestingly, the characteristic peak of metal–PMS* vanished in the Cu–ZIS_V_ + PMS + TC. The disappearance of the characteristic peak suggested that TC caused O─O bond breaking in side‐on metal–PMS*, interrupting the formation of peroxo‐species bonding to the surface metal sites, and that PMS was efficiently activated at the asymmetric Cu–S_V_–Zn site. The above results provide evidence of the interaction between the Cu–S_V_–Zn sites and O─O in side‐on PMS, highlighting the increased efficiency of PMS activation through polar charge transfer on asymmetric active centers.

**FIGURE 4:**
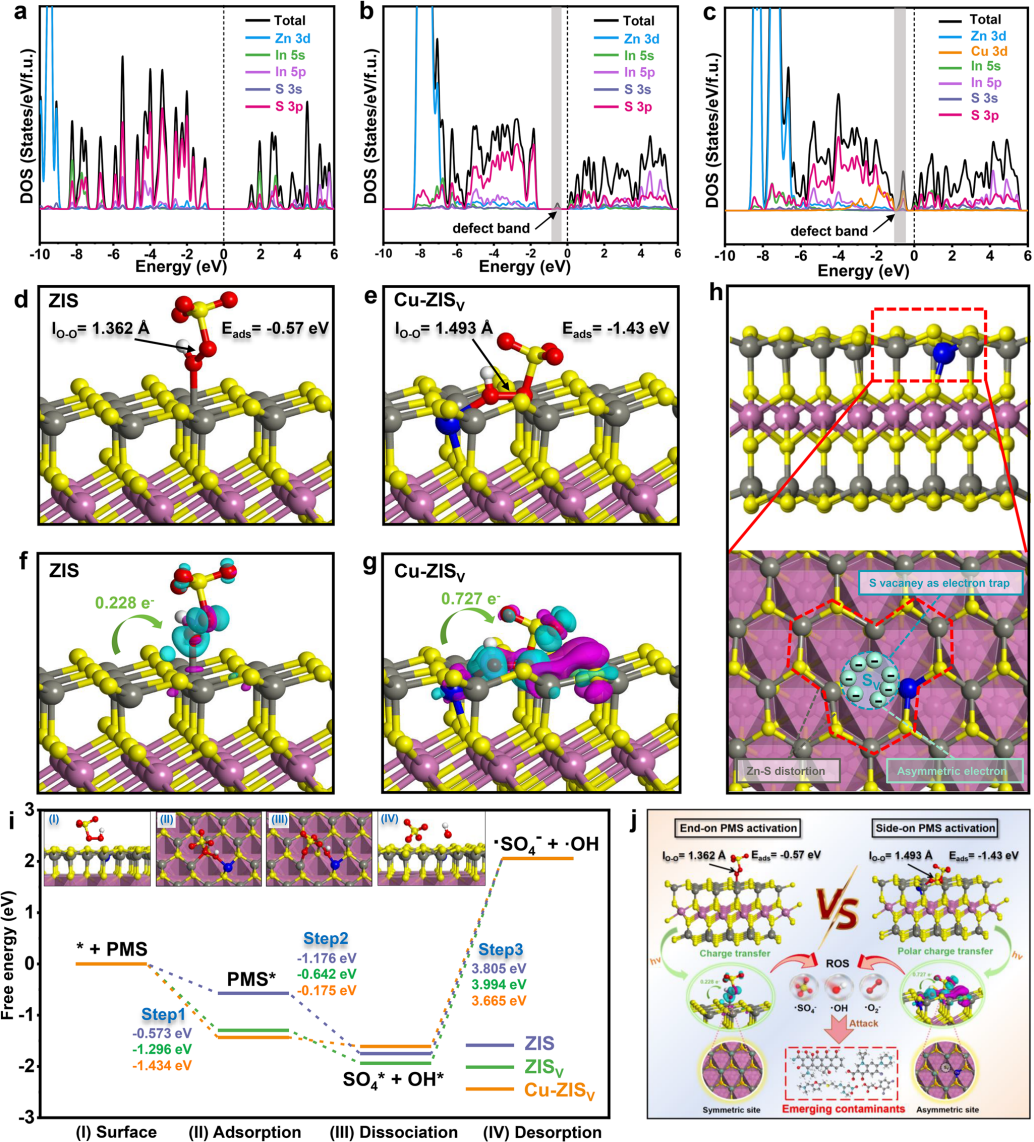
Density of states spectra of (a) ZIS, (b) ZIS_V_, and (c) Cu–ZIS_V_. Optimized adsorption configurations of PMS molecules on the surface of the (d) ZIS and (e) Cu–ZIS_V_ models. The difference in charge density and corresponding charge transfer for PMS adsorption on (f) ZIS and (g) Cu–ZIS_V_ were determined using the following equation: Δρ = ρ(total)—ρ(surface)—ρ(PMS). (h) Schematic of asymmetric Cu–S_V_–Zn sites over Cu–ZIS_V_ (Zn: black; In: purple; O: red; S: yellow; H: white). (i) Free energy profile and reaction pathway of PMS activation for the production of •SO_4_^−^ and •OH (the inset shows the corresponding intermediate structures and the energy barrier values corresponding to each step of different samples; the calculated intermediate energies are listed in Table S8). (j) A side‐on modulation enhancement mechanism for the Cu–ZIS_V_ + PMS + Vis system.

Density functional theory (DFT) calculations were performed to investigate the surface adsorption–activation of PMS over ZIS, ZIS_V_, and Cu–ZIS_V_ (Figure 4d,e; Figures S28 and S29 and Table S12). With respect to ZIS, the O─O bond of PMS was end‐on adsorbed at Zn–S–Zn sites, whereas side‐on adsorption was observed at Zn–S_V_–Zn and Cu–S_V_–Zn sites. The adsorption energies of PMS on the Zn–S–Zn, Zn–S_V_–Zn, and Cu–S_V_–Zn sites are −0.57, −1.29, and −1.43 eV, respectively, highlighting the excellent chemisorption reactivity of the Cu–ZIS_V_ photocatalyst. Significantly, the O─O bond lengths at these sites were calculated to be 1.362, 1.481, and 1.493 Å, respectively, suggesting that the self‐adaptive S vacancies are the basis for the creation of side‐on PMS*. The O─O bond in PMS with Cu–ZIS_V_ represented an elongation rate of 10.3%, indicating that the O─O bond was substantially weakened and prone to cleavage under side‐on PMS activation (Table S13). The S─O bond (peroxide side) in PMS with Cu–ZIS_V_ was shortened from 1.925 to 1.863 Å (a shrinkage rate of 3.2%), whereas the O─H bond was shortened from 1.073 to 1.019 Å (a shrinkage rate of 2.3%), suggesting that more electrons were injected into the O─O bond, promoting the side‐on activation of PMS to •OH/•SO_4_^−^ and inhibiting the formation of ^1^O_2_. The self‐adapting S vacancy optimizes the spatial environment for the simultaneous adsorption of dual oxygen atoms from O─O bonds in PMS. The polarized distribution and transfer mechanism of photogenerated electrons are improved by the asymmetric Cu–S_V_–Zn site, which capably adsorbs and stretches the O─O bond. The side‐on PMS* accelerates the PMS breakdown, resulting in a greatly enhanced production capacity of •SO_4_^−^ and •OH.

In conclusion, the S vacancy‐mediated side‐on activation modulated by S vacancies was confirmed in both ZIS_V_ and Cu–ZIS_V_, indicating the formation of an efficient side‐on photo‐Fenton‐like oxidation pathway. Furthermore, a more significant interaction appears to be established between side‐on PMS and Cu–ZIS_V_ than between side‐on PMS and ZIS_V_, likely because of the synergistic effect between side‐on PMS activation and the polar charge transfer of asymmetric active centers.

#### 2.3.3 Polar Charge Transfer and Interactions Between Cu–SV–Zn Sites and PMS

Density of states (DOS) spectral analysis for ZIS and ZIS_V_ revealed that the main contributions to the characteristic peaks arose from the S 3p orbital (Figure 4a–c). The emergence of a defect energy level (defect state) in ZIS_V_ lowered the excitation energy between the VB and CB, enhancing the conductivity and facilitating electron migration. Upon Cu doping, the Cu 3d orbital appeared near the Fermi energy level in Cu–ZIS_V_, causing a shift of the DOS characteristic peak toward the Fermi level. Compared with ZIS and ZIS_V_, Cu–ZIS_V_ resulted in a significantly strengthened defect state, which improved the absorption capabilities of visible light and enhanced the separation efficiency of the photogenerated electron–hole pairs [50, 51]. Importantly, Cu‐doping drives the adaptation of S vacancies, resulting in increased electron density near the Fermi energy level. The higher charge density and closer proximity to the Fermi energy markedly improved the polar distribution of photogenerated electrons at the Cu–S_V_–Zn site, thus increasing the chemical reactivity and charge transfer efficiency between the catalysts and PMS.

The polar charge transfer at the Cu–S_V_–Zn site during PMS adsorption–activation was further clarified by the charge density difference (CDD) (Figure 4f–g; Figure S30 and Table S14). The purple region represents electron depletion, whereas the blue region represents electron accumulation. Following PMS adsorption, electron depletion was localized on Cu–ZIS_V_, while electron accumulation was concentrated on PMS, presenting direct evidence for polar charge transfer to PMS via the Cu–S_V_–Zn sites. Moreover, the calculation of the number of electrons transferred from the catalyst to PMS revealed that PMS receives more charge from Cu–ZIS_V_ (0.727 e^−^) than from ZIS (0.228 e^−^) or ZIS_V_ (0.652 e^−^). The presence of asymmetric sites and S vacancies diminished the internal electric field, hindering the migration of photogenerated electrons toward the In atom layer (Figure 4h). These S vacancies subsequently acted as electron traps to enrich the photogenerated electrons, which are involved in the activation of PMS. Together, these results theoretically confirmed that the Cu–S_V_–Zn sites facilitate effective side‐on activation and rapid polar electron transfer.

The mechanism underlying PMS activation and the relationship between the active site structure and reaction performance were studied via Gibbs free energy calculations. The intermediate structure and energy distribution throughout the reaction process are displayed in Figure 4i–j and Table S15. The reaction process of PMS decomposition over ZIS_V_ and Cu–ZIS_V_ follows three steps:

Step 1 (Surface → Adsorption): * + PMS → end‐on PMS*/side‐on PMS*
Step 2 (Adsorption → Dissociation): end‐on PMS*/side‐on PMS* → OH* + SO4*
Step 3 (Dissociation → Desorption): OH* + SO4* → * + •OH + •SO4−

where * represents the (001) surfaces of ZIS, ZIS_V_, and Cu–ZIS_V_, and species* denotes the corresponding surface‐bound species complex. The blue, green, and orange pathways represent the activation mechanisms of the Zn–S–Zn, Zn–S_V_–Zn, and Cu–S_V_–Zn sites, respectively. In step 1, PMS activation initiated an adsorption process, where the adsorption energy at Cu–S_V_–Zn sites negatively correlated with the PMS adsorption efficiency. Cu–S_V_–Zn exhibited higher adsorption efficiency than Zn–S–Zn and Zn–S_V_–Zn. Step 2 involved the dissociation process of PMS activation, where the energy changed from end‐on PMS*/side‐on PMS* to OH* and SO_4_*, reflecting the generation of •OH and •SO_4_^−^. Notably, the Cu–S_V_–Zn sites had a dissociation energy of 0.175 eV for side‐on PMS* activation, which was substantially lower than that of the Zn–S–Zn sites (1.176 eV) and Zn–S_V_–Zn sites (0.642 eV). This distinction arises from the synergistic effect between Cu single atoms and self‐adapting S vacancies: Cu single atoms modulate the polar electronic density of the active center, whereas self‐adapting S vacancies tune the local coordination environment, collectively driving O−O bond breaking in side‐on PMS*. In step 3, the desorption of OH* and SO_4_* to form •OH and •SO_4_^−^ was the primary process that limited the reaction rate between PMS and the catalyst. At the Cu–S_V_–Zn sites (3.665 eV), the desorption energies of •OH and •SO_4_^−^ were lower than those at the Zn–Sv–Zn sites (3.995 eV) and Zn–S–Zn sites (3.805 eV), illustrating that the asymmetric Cu–S_V_–Zn sites could produce more •OH and •SO_4_^−^ through side‐on PMS* activation. On the basis of the above analysis, the side‐on PMS* induced by the asymmetric Cu–S_V_–Zn sites reinforced the interaction between PMS and the photocatalyst, promoting the polarity transfer of photogenerated charge, which can improve the degradation efficiency of the ECs.

### 2.4 Practical Application Potential of Side‐On Photo‐Fenton‐Like Processes

**FIGURE 5:**
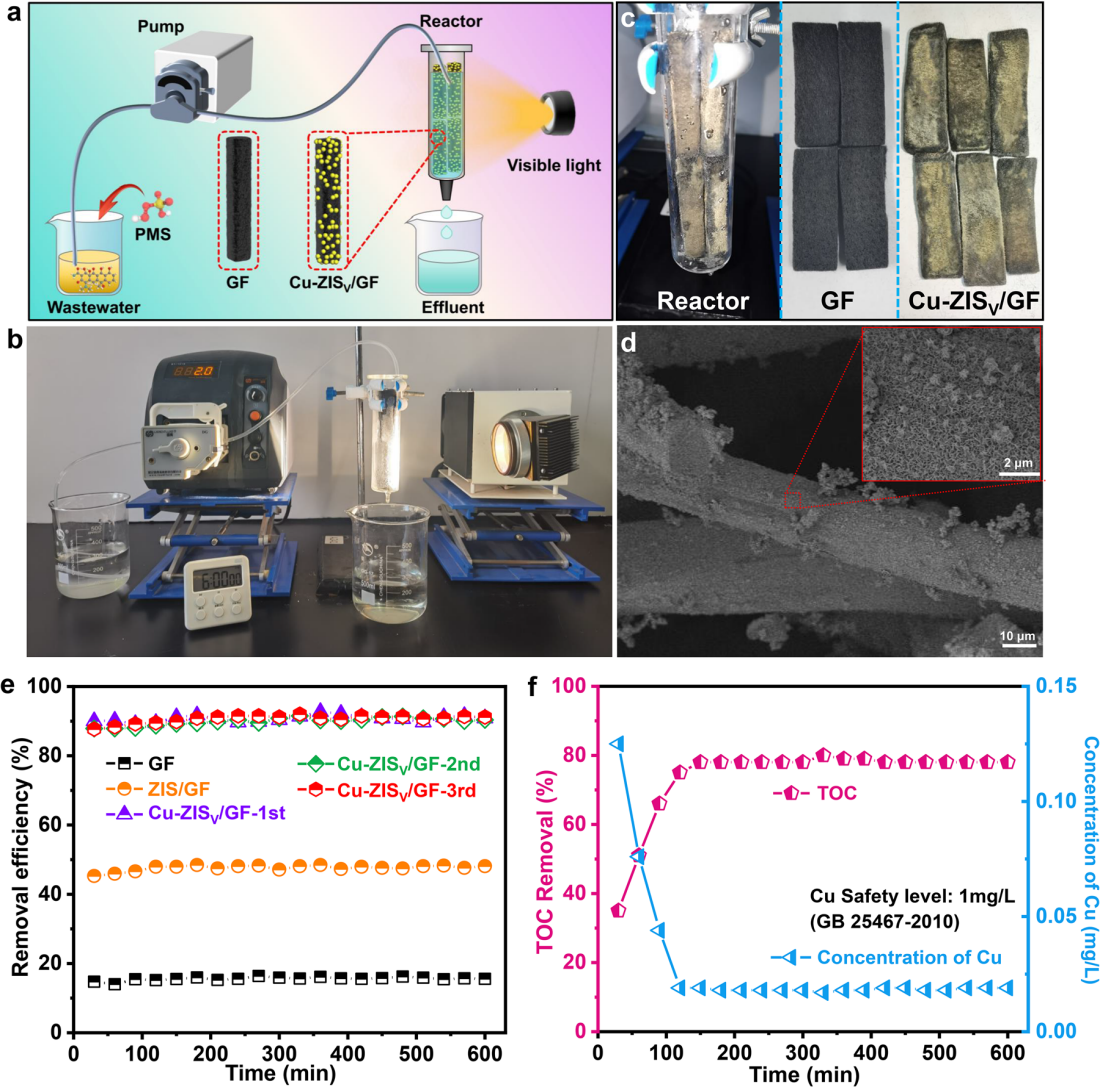
(a) Schematic of the small‐scale wastewater treatment equipment process. (b) Photograph of the wastewater treatment experimental equipment. (c) Photograph of the reactor, GF, and Cu–ZIS_V_/GF. (d) SEM image of Cu–ZIS_V_/GF. (e) TC removal efficiency of GF, ZIS/GF, and Cu–ZIS_V_/GF. (f) TOC removal rate and concentration of Cu over time.

To evaluate the practical application potential of the side‐on photo‐Fenton‐like process, a continuous‐flow reactor was constructed in which Cu–ZIS_V_ was immobilized on a graphite melt (Cu–ZIS_V_/GF) via an in situ immobilization method (Figure 5a,b). Owing to the significant specific surface area and complicated porous structure of GF, the catalyst was stably attached, ensuring robust retention throughout the wastewater treatment process (Figure 5c). Cu–ZIS_V_ was observed in the form of nanosheets affixed to the GF surface in the SEM images (Figure 5d). To assess the performance of Cu–ZIS_V_/GF, the concentration of TC introduced into the wastewater served as an indicator to evaluate organic removal efficiency. After three continuous operational cycles, the Cu–ZIS_V_/GF reached a photocatalytic degradation efficiency for TC of more than 91%, and a stable flux rate was maintained (Figure 5e; Figure S31). Notably, its performance outperformed that of ZIS/GF owing to the synergistic effect of polar charge transfer and side‐on PMS*. Moreover, the Cu–ZIS_V_/GF system can maintain a satisfactory TOC removal rate, and the concentration of Cu ion leaching is lower than the discharge standard of China (1.0 mg/L, GB 25467‐2010) (Figure 5f). The residual TOC is attributed mainly to the relatively stable intermediates P10, P11, P12, P13, and P14 (Figure S21 and Table S9). Small‐molecular carboxylic acids (P10 and P12) were resistant to ROS attack owing to their high oxidation state and low reactivity with •OH/•SO_4_^−^, leading to high activation energy and the need for longer continuous‐flow residence times for further oxidation. These continuous‐flow results indicated that the polar charge transfer and side‐on photo‐Fenton‐like oxidation pathway offered a reliable guarantee of both efficiency and stability for potential practical antibiotic wastewater treatment.

## 3 Conclusion

In this study, the asymmetric active center model of Cu–ZIS_V_ was employed to systematically investigate the relationship between the photocatalyst structure and PMS activation kinetics, highlighting the modulation mechanism of polar charge transfer and self‐adapting S vacancies on side‐on photo‐Fenton‐like oxidation pathways. The incorporation of Cu atoms triggered local structural self‐adaptive to form asymmetric Cu–S_V_–Zn sites, which resulted in rapid interfacial charge transfer through polar distribution, thus inducing side‐on PMS activation modulation. Through side‐on PMS activation, the asymmetric active center decreased the free energy barrier and elongated the O─O bond in PMS, supporting a more efficient interfacial reaction. The side‐on PMS* and polar charge transfer synergistically contributed to the superior catalytic potential of Cu–ZIS_V_ in the photo‐Fenton‐like system. Furthermore, the Cu–ZIS_V_ + PMS + Vis system illustrated remarkable environmental adaptability, safety, and practical feasibility in the continuous‐flow treatment of antibiotic wastewater, which was attributed to the substantial increase in catalyst stability via local structural self‐adaptation. Overall, this study offers a novel atomic‐scale self‐adaptive strategy for asymmetric site formation, highlighting the remarkable potential of self‐adapting modulation to enhance side‐on PMS activation in practical photo‐Fenton‐like process applications.

## 4 Experimental Section

### 4.1 Chemicals

All the chemicals and reagents used in this work are listed in Text S1. Unless otherwise specified, all the chemicals and reagents were of at least analytical grade and were used without further purification.

### 4.2 Catalyst Preparation

Preparation of Cu–Zn_3_In_2_S_6_ with S vacancies: A solution containing 3 mm ZnSO_4_•7H_2_O, 2 mm InCl_3_•4H_2_O, and 12 mm thioacetamide (TAA) was dissolved in 70 mL of ultrapure water and stirred continuously for approximately 30 min. Subsequently, 1.8 mm cetyltrimethyl ammonium bromide (CTAB) and various amounts of CuSO_4_•5H_2_O (2.4, 7.2, 12, 16.8, 24.0, 36.0, 48.0 mg) were slowly added, followed by vigorous sonication and stirring for 2 h, resulting in the formation of self‐adapting S vacancies via partial substitution of Zn atoms by Cu.

The resulting solution was transferred to a 100 mL Teflon‐lined stainless‐steel autoclave. The autoclave was maintained at 160°C for 12 h to form stable asymmetric active sites through local structural self‐adaptation. Afterward, the autoclave was permitted to cool passively until it reached ambient temperature. The precipitate was processed by washing six times (all final products were denoted as x%Cu–ZIS_V_, where x is the mass ratio of Cu to ZIS_V_).

In addition, details on the synthesis of Zn_3_In_2_S_6_ (ZIS) and Zn_3_In_2_S_6_ with S vacancies (ZIS_V_) are provided in Text S2.

### 4.3 Experimental Procedure and Characterization

The details of the experimental procedures, theoretical computations, and characterization, including in situ Raman spectroscopy, X‐ray absorption fine structure (XAFS) spectroscopy, photoelectrochemical analysis, and electron spin resonance (ESR) spectroscopy, were shown in Texts S3–S11.

## Conflicts of Interest

The authors declare no conflicts of interest.

## Supporting information




**Supporting File**: advs74376‐sup‐0001‐SuppMat.docx

## Data Availability

The data that support the findings of this study are available from the corresponding author upon reasonable request.
