# Supplementary material for: Asymmetric Active Center Triggers Side‐On Photo‐Fenton‐Like Reaction Through Polar Charge Transfer and Self‐Adapting Sulfur Vacancies
Source: Adv Sci (Weinh). 2026 Feb 10;13(23):e17517. doi: 10.1002/advs.202517517 (PMC13104082; doi:10.1002/advs.202517517)
Supplement: Supplementary file 1 — Supporting File: advs74376‐sup‐0001‐SuppMat.docx [file ADVS-13-e17517-s001.docx]

**Supporting Information**

**Asymmetric active center triggers side-on photo-Fenton-like reaction through polar charge transfer and self-adapting sulfur vacancies**

Jiaqi Hu^a^, Xiaoyuan Zhang^a^, Yufei Gao^a^, Jianyi Liu^a^, Xu He^b^, Yu Liu^c^, Jinfeng Lu^a, *^, Jun Ma^b^

*^a^* Key Laboratory of Pollution Processes and Environmental Criteria of Ministry of Education, Tianjin Key Laboratory of Environmental Technology for Complex Trans-Media Pollution, College of Environmental Science and Engineering of Nankai University, Tianjin 300350, China

*^b^* State Key Laboratory of Urban Water Resource and Environment, Harbin Institute of Technology, Harbin 150090, China

^*^ Corresponding author’s e-mail: lujinfeng@nankai.edu.cn (J. F. Lu)

**This Supporting Information provides:**

Total number of pages: 69

Total number of Texts: 11

Total number of Figures: 31

Total number of Tables: 15**Text S1. Materials**

Copper nitrate (CuSO_4_•5H_2_O, 99%, purity), zinc sulfate (ZnSO_4_•7H_2_O, 99%, purity), indium chloride (InCl_3_•4H_2_O, 99%, purity), thiacetamide (TAA, 99.0%, purity) L-histidine (L-H, 99%, purity) and urea were purchased from Aladdin Chemistry Co. Ltd (Shanghai, China). TC (98%, purity), CIP (98%, purity) and MB (98%, purity) were supplied by Dalian Meilun Biological Technology Co. Ltd. (Tianjin, China). Isopropyl alcohol (IPA, 98%, purity), potassium dichromate (K_2_Cr_2_O_7_, 98%, purity), methanol (MeOH 98%, purity), and ethanol absolute (99%, purity) were provided by Sinopharm Chemical Reagent Co., Ltd (Shanghai, China). Sorbic acid (97%), nitro-blue tetrazolium (NBT, 98%), furfuryl alcohol (FFA, 98%), benzoic acid (BA, 98%), nitrobenzene (NB, 98%), 2,4-D (98%, purity), RAN (97%, purity) and cetyltrimethyl Ammonium Bromide (CTAB, 98%) were purchased from Shanghai Macklin Biochemical Co., Ltd. Other chemicals such as ethylenediaminete tetraacetic acid disodium salt (EDTA-2Na, 98%, purity), and benzoquinone (BQ, 98%, purity) were obtained from Tianjin Concord Technology Co., Ltd. (Tianjin, China). All chemicals utilized were of analytical grade and employed without further purification.

**Text S2. Methods**

**Preparation of Zn_3_In_2_S_6_**

A solution containing 3 mM ZnSO_4_•7H_2_O, 2 mM InCl_3_•4H_2_O, and 12 mM Thioacetamide (TAA) was dissolved in 70 ml of ultra-pure water while being continuously stirred for approximately 30 min. Subsequently, the resulting solution was put into a 100 mL stainless-steel autoclave, which had been lined with Teflon. The autoclave was maintained at 160 ℃ for 12 h. Then, the autoclave was permitted to cool passively until it reached ambient temperature. The precipitate was processed by washing six times (denoted as ZIS).

**Preparation of Zn_3_In_2_S_6_ with S vacancies**

A solution containing 3 mM ZnSO_4_•7H_2_O, 2 mM InCl_3_•4H_2_O and 12 mM TAA was dissolved in 70 ml of ultra-pure water while being continuously stirred for approximately 30 min. Subsequently, 1.8 mM of Cetyltrimethyl Ammonium Bromide (CTAB) was added slowly with stirring. The resulting solution was put into a 100 mL stainless-steel autoclave, which had been lined with Teflon. The autoclave was maintained at 160 ℃ for 12 h. Then, the autoclave was permitted to cool passively until it reached ambient temperature. The precipitate was processed by washing six times (denoted as ZIS_V_).

**Preparation of Cu-ZIS_V_ loaded with Graphite Felt (Cu-ZIS_V_/GF)**

A solution containing 3 mM ZnSO_4_•7H_2_O, 2 mM InCl_3_•4H_2_O and 12 mM of TAA were dissolved in 70 ml of ultra-pure water while being continuously stirred for approximately 30 min. Subsequently, 1.8 mM of Cetyltrimethyl Ammonium Bromide (CTAB) and 2.4, 7.2, 12, 17, 24, 36, 48 mg of CuSO_4_•5H_2_O were added slowly with sonicating and stirring vigorously for 2 h, resulting in the formation of self-adapting S vacancies induced by Cu replacing Zn atoms. The solution and Graphite Felt bulk was put into a 100 mL stainless-steel autoclave, which had been lined with Teflon. The autoclave was maintained at 160 ℃ for 12 h. Then, the autoclave was permitted to cool passively until it reached ambient temperature to obtain Cu-ZIS_V_ loaded with Graphite Felt (denoted as Cu-ZIS_V_/GF).

**Text S3. Experimental characterizations**

For high-angle annular dark-field scanning transmission electron microscopy (HAADF-STEM, JEM-ARM200F, Japan) characterization, an FEI Titan Themis G1 equipped with dual aberration correctors and four energy-dispersive X-ray spectroscopy (EDS) detectors were used. Lattice images were carried out using a JEM-2100F transmission electron microscope (Jeol, Tokyo, Japan). Chemical environment information was gathered by the use of ESCALAB250 photoelectron spectrometer (Thermo Fisher Scientific, Waltham, MA, USA) for X-ray photoelectron spectroscopy (XPS) and inductively coupled plasma mass spectrometry (ICP-MS, Elan drc-e, USA). An analyzer developed by Shimadzu Corp. of Kyoto, Japan, known as the TOC-5000A TOC/TN, was used to measure the total organic carbon (TOC). Mobile phases A and B, consisting of 0.1 percent formic acid and 80/20% acetonitrile, respectively, were used to accomplish chromatographic separation using an Agilent Eclipse plus C18 column. To investigate pollutant byproducts, a Thermo Fisher Orbitrap Fusion LC-MS system was utilized in conjunction with electrospray positive ionization multiple-reaction monitoring mode. Photoluminescence spectra of the samples were investigated using a steady/transient spectrophotometer (Xelamp-920-equipped FLS920P, Edinburgh Instruments, Livingston, UK). An X-ray diffraction (XRD; Ultima IV, Rigaku, Tokyo, Japan) examination was performed to identify the crystal structure. The optical characteristics of the samples were ascertained using ultraviolet-visible diffuse reflection spectroscopy (UV-vis DRS) with a Shimadzu UV-3600 instrument.

**Text S4. In-situ Raman**

Raman analysis was done with a confocal Raman microscope (CRM) (Alpha300R, WITec GmbH, Germany) equipped with a TEM single-frequency laser (λ=532 nm, laser power=40 mW, WITec GmbH, Germany). The laser light was focused through a 100x oil immersion objective (numerical aperture=0.9) (Carl Zeiss, Germany) onto the sample and the backscattered Raman signal directed through an optic multifibre (50 um diameter) to a spectrometer (UHTS 300 WITec, Germany) (300 g mm^-1^ grating) and detected by the CCD camera (Andor DU401 BV, Belfast, North Ireland). On the selected areas (e.g., 30 um × 20 um) on the sample every 0.5 um a full wavenumber range (50-4000 cm^-1^) Raman spectrum was acquired with an integration time of 1s. The Control Five (WITec GmbH, Germany) acquisition software was used for the Raman measurements set up and Project Five (WITec GmbH, Germany) to reconstruct Raman images based on the integral band of the ester group at 1734 cm^-1^ and the hydroxyl groups at 3400 cm^-1^.

**Text S5. X-ray absorption spectroscopy (XAS) data collection and analysis**

The X-ray absorption fine structure spectra (Cu K-edge) were collected at 1W1B station in Beijing Synchrotron Radiation Facility (BSRF). The storage rings of BSRF were operated at 2.5 GeV with an average current of 250 mA. Using a Si (111) double-crystal monochromator, the data collection was carried out in transmission/fluorescence mode using ionization chamber. All spectra were collected under ambient conditions. The acquired EXAFS data were processed according to the standard procedures using the ATHENA module implemented in the IFEFFIT software packages. The k^3^-weighted EXAFS spectra were obtained by subtracting the post-edge background from the overall absorption and then normalizing with respect to the edge-jump step. Subsequently, k^3^-weighted χ(k) data of Cu K-edge were Fourier transformed to real (R) space using a Hanning window (dk=1.0 Å^-1^) to separate the EXAFS contributions from different coordination shells. To obtain the quantitative structural parameters around central atoms, least-squares curve parameter fitting was performed using the ARTEMIS module of the IFEFFIT software packages.

**Text S6. Photoelectrochemical measurements.**

A uniform suspension of material (20 mg) was prepared with nafion (20 mL) and ethanol (1 mL) and ultrasonically agitated for 30 min. Then, the surface of indium tin oxide conductive glass was filled with the suspension. The conductive glass could be used for further testing after ethanol was volatilized completely. Photoelectrochemical was measured using a CHI760E electrochemical workstation (CH Instruments, Inc., Austin, TX, USA). The light source was the same as that for the degradation process. In the three-electrode system, Ag/AgCl, Pt sheet, and conductive glass served as reference electrode, counter electrode, and photoanode, respectively.

**Text S7. Electron spin resonance (ESR) test method**

The 30 μl of the sample and 30 μl of DMPO (dissolved in 100 mM deionized water/methanol as a solvent) were mixed thoroughly and then absorbed a specific quantity of the mixture using a capillary. Cover a quartz tube and place it in the sample cavity for testing the •OH + •SO_4_^-^ / •O_2_^-^. The 200 μl of sample and 2 μl TEMPO (100 mM) were mixed evenly. After that, a certain amount of mixture has been absorbed and covered with a quartz tube to test the signal of ^1^O_2_.

**Text S8. Experimental procedure.**

Under visible-light irradiation, the photocatalytic activity of the Cu-ZIS_V_ + PMS + Vis system was assessed to determine ECs abatement. The light source used was a CEL-HXF300 Xenon arc lamp, which produced 180 mW/cm^2^ light intensity at a current of 15 A.

In a typical test, 20 mg of catalyst was added to 50 mL of pollutants containing 1.5 mM PMS in a 50 mL beaker. After attaining to adsorption-desorption equilibrium in dark condition, visible-light was intercepted using a 420 nm filter. At predetermined time intervals, 3 mL aliquots were filtered through a 0.22 μm nylon membrane and analyzed using a UV-visible spectrophotometer to determine contaminant concentrations. The degradation kinetics were evaluated using equation:

$\ln\left( {\text{C}_{\text{t}}}/{\text{C}_{\text{0}}} \right)\text{=}\text{ }\text{-}\text{k}_{\text{obs}}\text{ × }$*t*

where *k*_obs_ is the apparent first-order kinetic constant, *C*_t_ and *C*_0_ are the TC concentrations at reaction time (*t*) and initial state (*t*_0_), respectively.

**Text S9. Theoretical calculation**

The Vienna Ab-initio Simulation Package (VASP) was used to conduct spin-polarized first-principle calculations based on the density functional theory (DFT).^[1,2]^ The electronic exchange and correlation effects were described using the Perdew−Burke−Ernzerhof (PBE) functional within the framework of the generalized gradient approximation (GGA).^[3-5]^ The G-centered k-point and Methfessel-Paxton electrical smearing methods were used to integrate the Brillouin zone throughout the geometric optimization process. The simulation was conducted using a fixed cutoff energy of 500 electron volts (eV) for all calculations. These parameters guarantee that the total energies will converge to within 1 meV per atom. The process of structure relaxing continued until the forces acting on the atoms were below 1 meV Å ^-1^ and the overall stress tensor was within 0.01 GPa of the desired value. In all computations, the DFT-D2 Van der Waals adjustment by Grimme was also taken into account.^[6]^

The Cu atoms substituted for the Zn atoms and produced self-adapting S vacancies in Zn_3_In_2_S_6_. Based on this, we constructed Zn_3_In_2_S_6_ model with Cu-doping and S vacancies. In Figure S28a, all Zn atom sites in the pristine Zn₃In₂S₆ were equivalent, and the vacancies formed by the removal of sulfur atoms were also equivalent (Figure S28b). After the construction of S vacancies (Figure S28c), the substitution of Zn atoms at 2, 4, and 6 sites with Cu atoms resulted in equivalent Cu-S_3_ with unsaturated coordination, which possessed the same catalytic effect. In contrast, the substitution of Zn atoms at 1, 3, and 5 sites with Cu atoms led to the formation of Cu-S_4_ with saturated coordination that lacked catalytic activity, thus the substitution of Zn atoms at 1, 3, and 5 sites with Cu atoms was excluded from consideration.

The calculation of adsorption energy was performed under vacuum condition to compare the relative magnitudes of adsorption effect between different active sites and PMS. Although the absolute value of the catalyst's adsorption energy became more positive under the solvation effect (approximately 0.27 eV, this is expected due to the shielding effect of water), the relative trends and core conclusions remain unchanged (for example, [species A] still exhibits stronger adsorption than [species B]), and the most stable adsorption configuration of the catalyst also remained consistent.^[7-10]^

**Text S10. Adsorption-desorption equilibrium experiment**

The adsorption of the samples was tested with different pollutants under dark condition. Briefly, 20 mg catalyst was dispersed into 50 mL solution with 20 ppm TC, which was then stirred to reach an adsorption-desorption equilibrium. At predetermined intervals of exposure, 3 ml of the suspension was sampled and filtered through a 0.22 μm nylon membrane for further measurements. In addition, the photocatalytic degradation reaction conditions were as follows: [catalyst] = 0.4 g·L^-1^, [TC] = 20 mg·L^-1^ (50 mL), [PMS] = 1.5 mM, [Ethylenediaminetetraacetic acid disodium salt (EDTA-2Na)] = 1 mM, [Methanol (MeOH)] = 0.5 mM, [L-histidine (L-H)] = 0.1 mM, [Potassium dichromate (K_2_CrO_7_)] = 0.05 mM, [Benzoquinone (BQ)] = 1 mM, and [Tert-Butano (TBA)] = 0.5 mM.

**Text S11. Continuous-flow experiment**

In order to assess the practical feasibility of Cu-ZIS_V_ + PMS + Vis system, a continuous-flow reactor comprising Cu-ZIS_V_ loaded with Graphite Felt (Cu-ZIS_V_/GF) was utilized for further evaluation. The continuous-flow experiments used TC as a target contaminant for three cycles and each cycle lasted 600 min without the need to wash the catalysts. The light source was a CEL-HXF300 Xenon arc lamp, which produced 180 mW/cm^2^ light intensity at a current of 15 A. Visible light was intercepted using a 420 nm filter. Pollutant removal effects were observed at predefined intervals using UV-vis spectrophotometers.

**2. Supporting Figures**


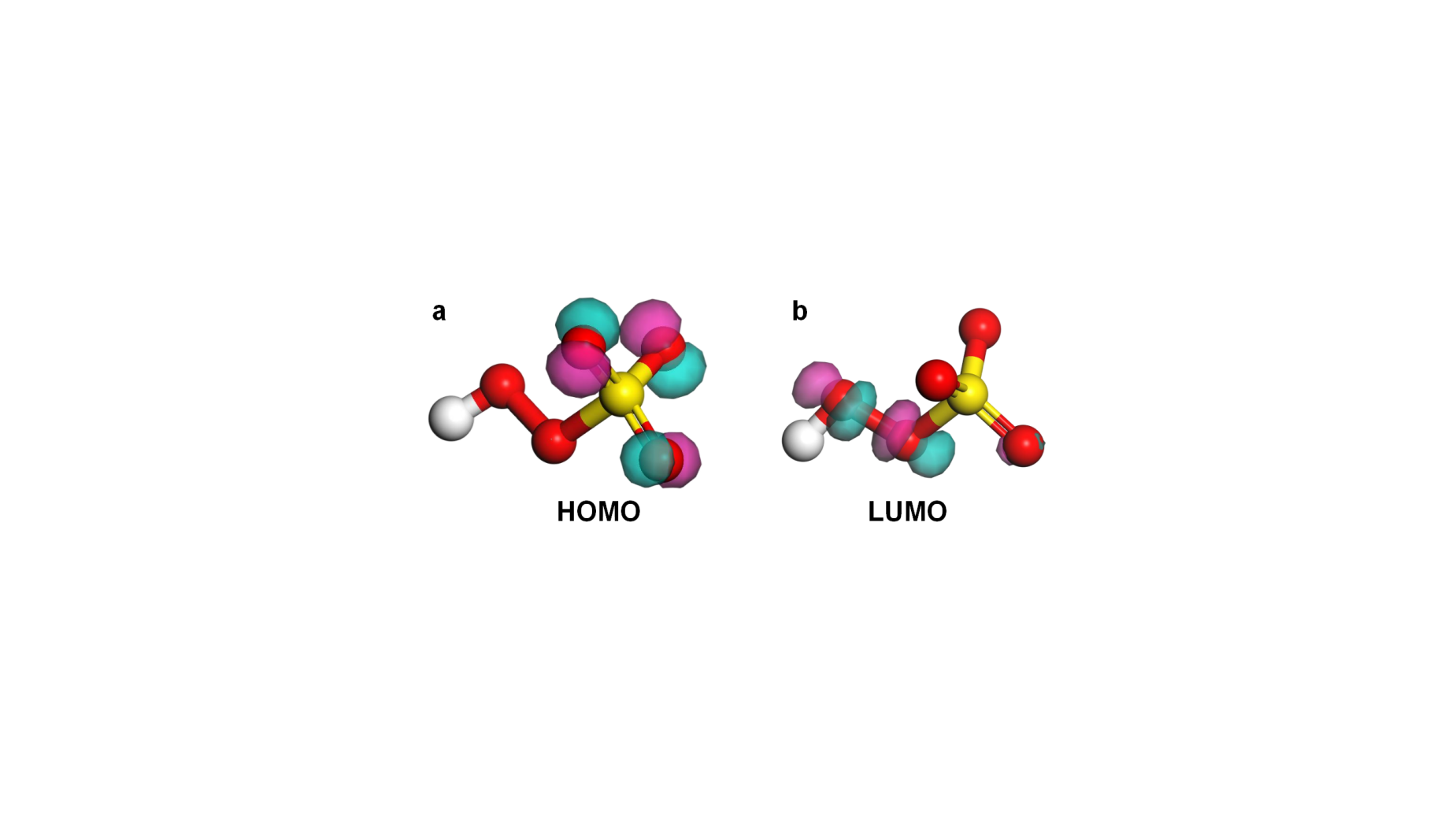


**Figure S1.** The charge distribution of (a) HOMO and (b) LUMO of PMS by photoexcitation.

**
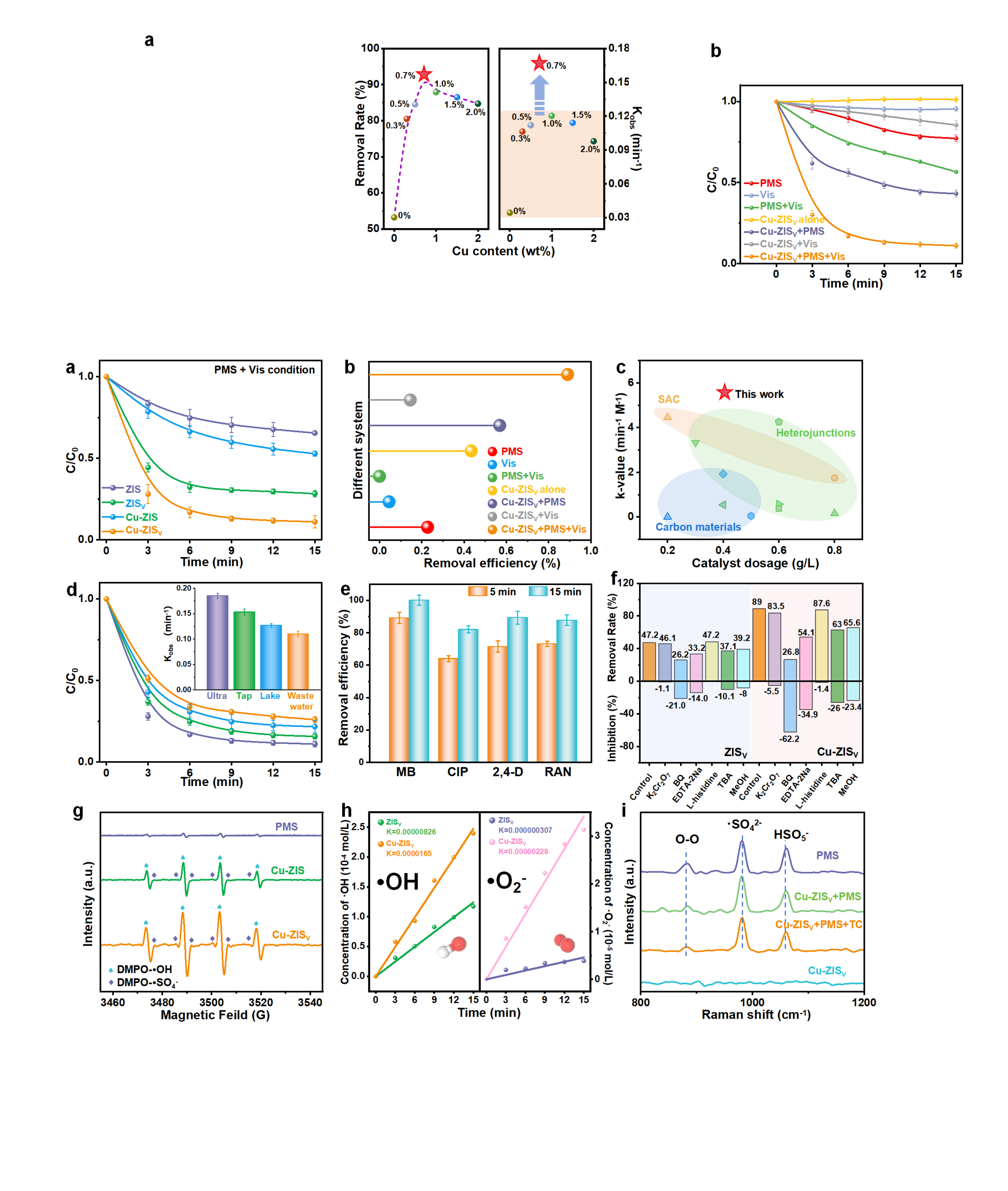
**

**Figure S2.** (a) First-order rate constants and TC degradation using x%Cu-ZIS_V_ with varying Cu concentrate.

**
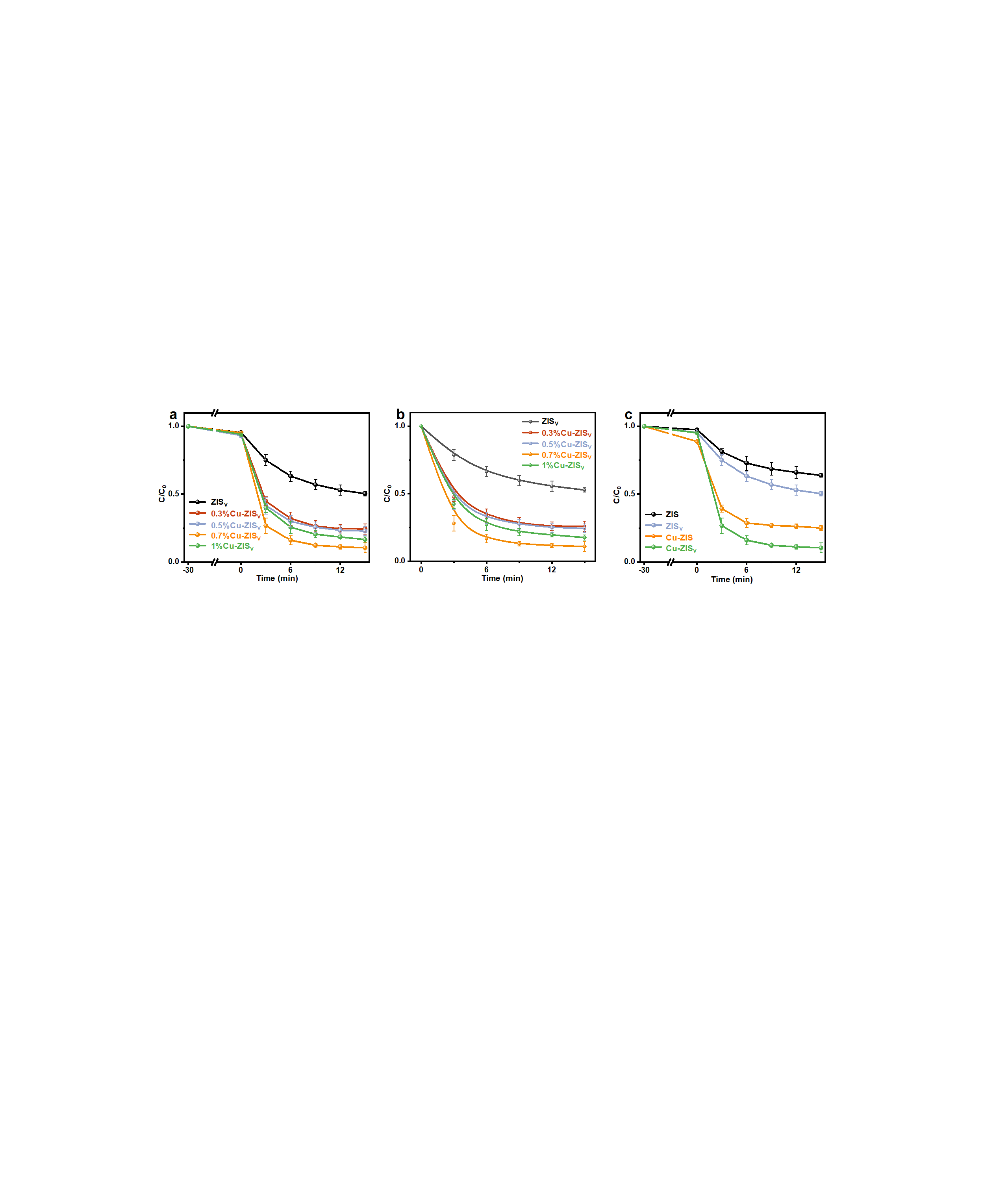
**

**Figure S3.** (a-b) Degradation efficiency of TC by x% Cu-ZIS_V_ with different Cu loadings.

**
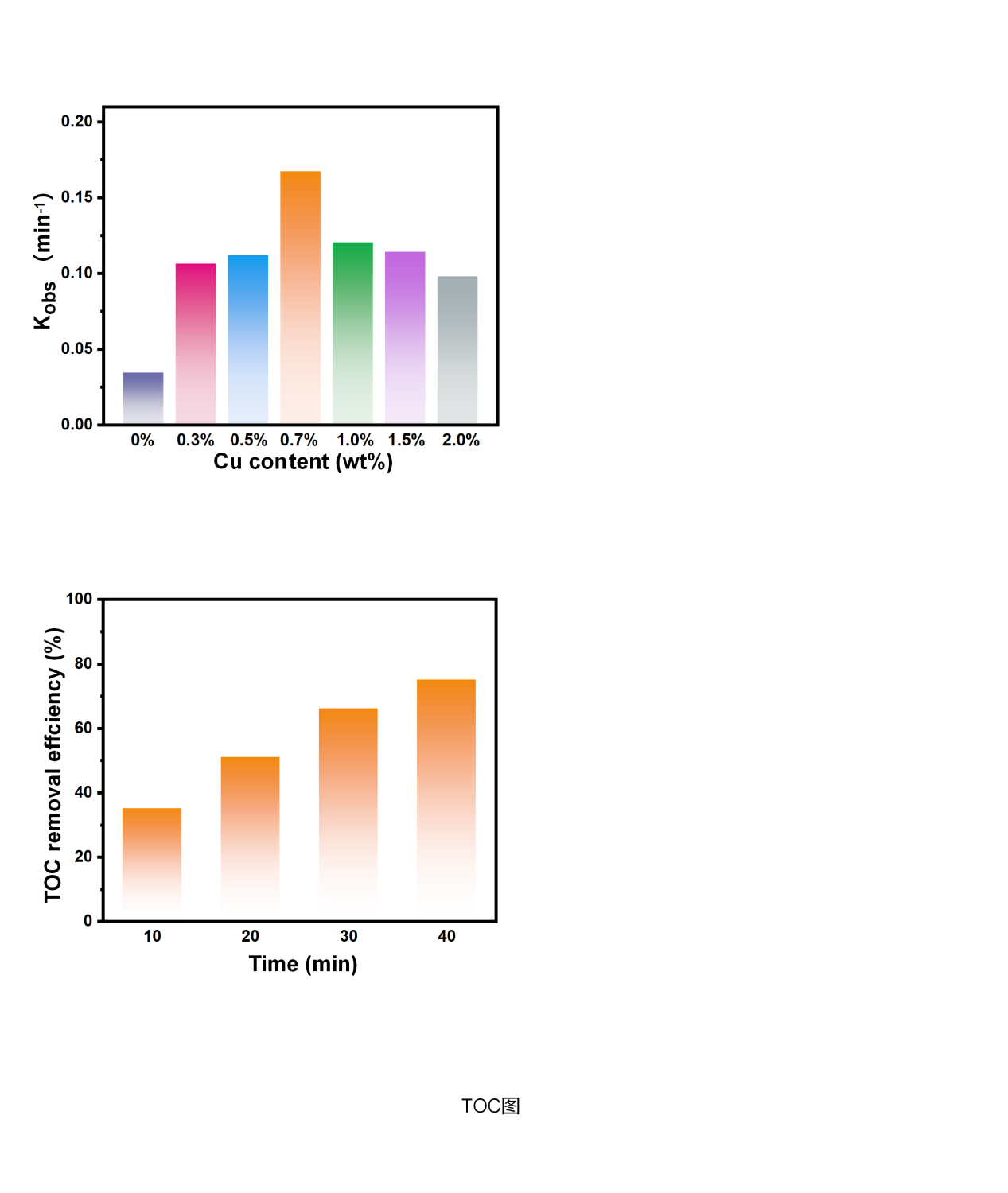
**

**Figure S4.** First-order rate constants of x% Cu-ZIS_V_ degraded TC.

**
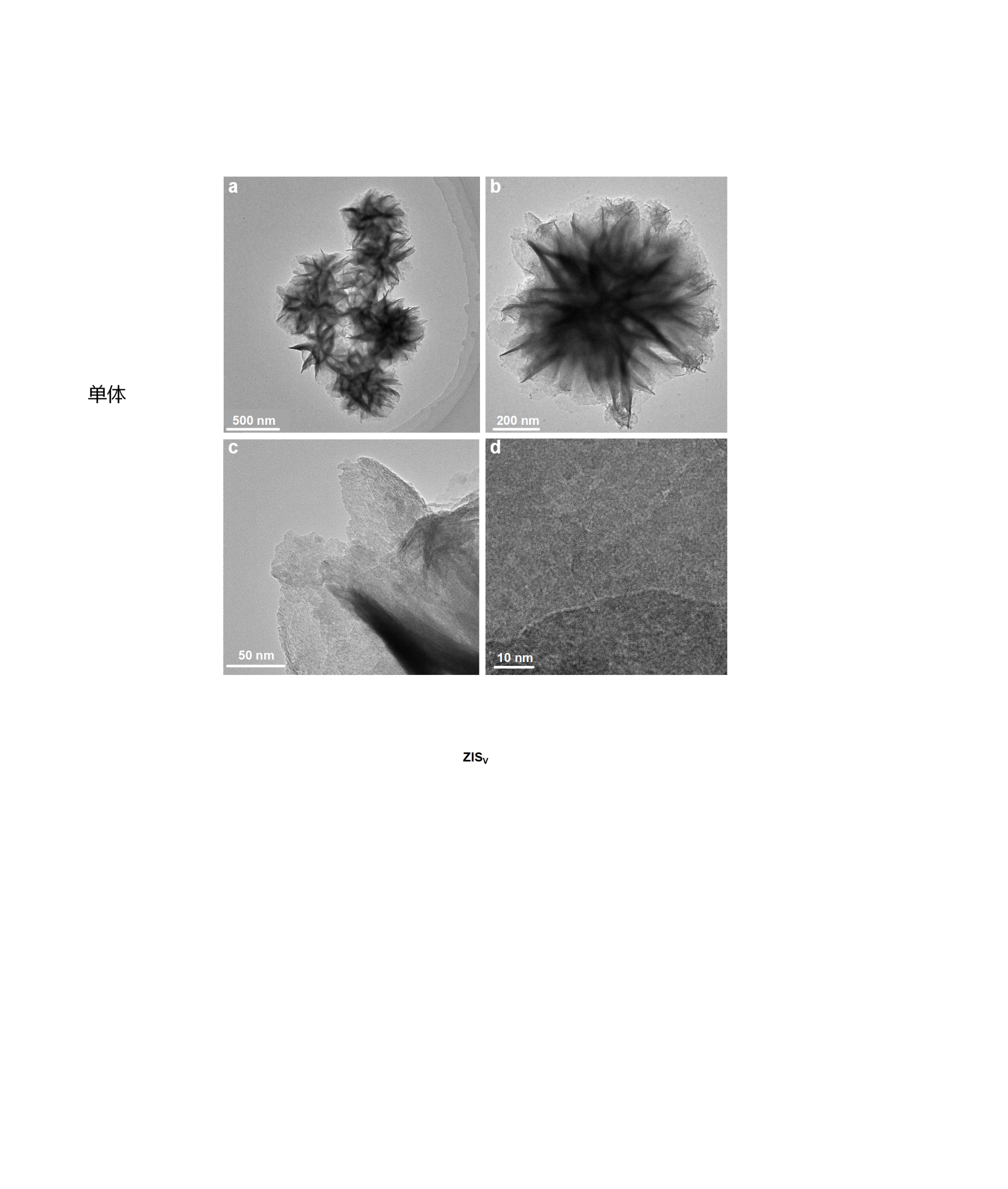
**

**Figure S5.** (a-d) TEM images of ZIS_V_.

**
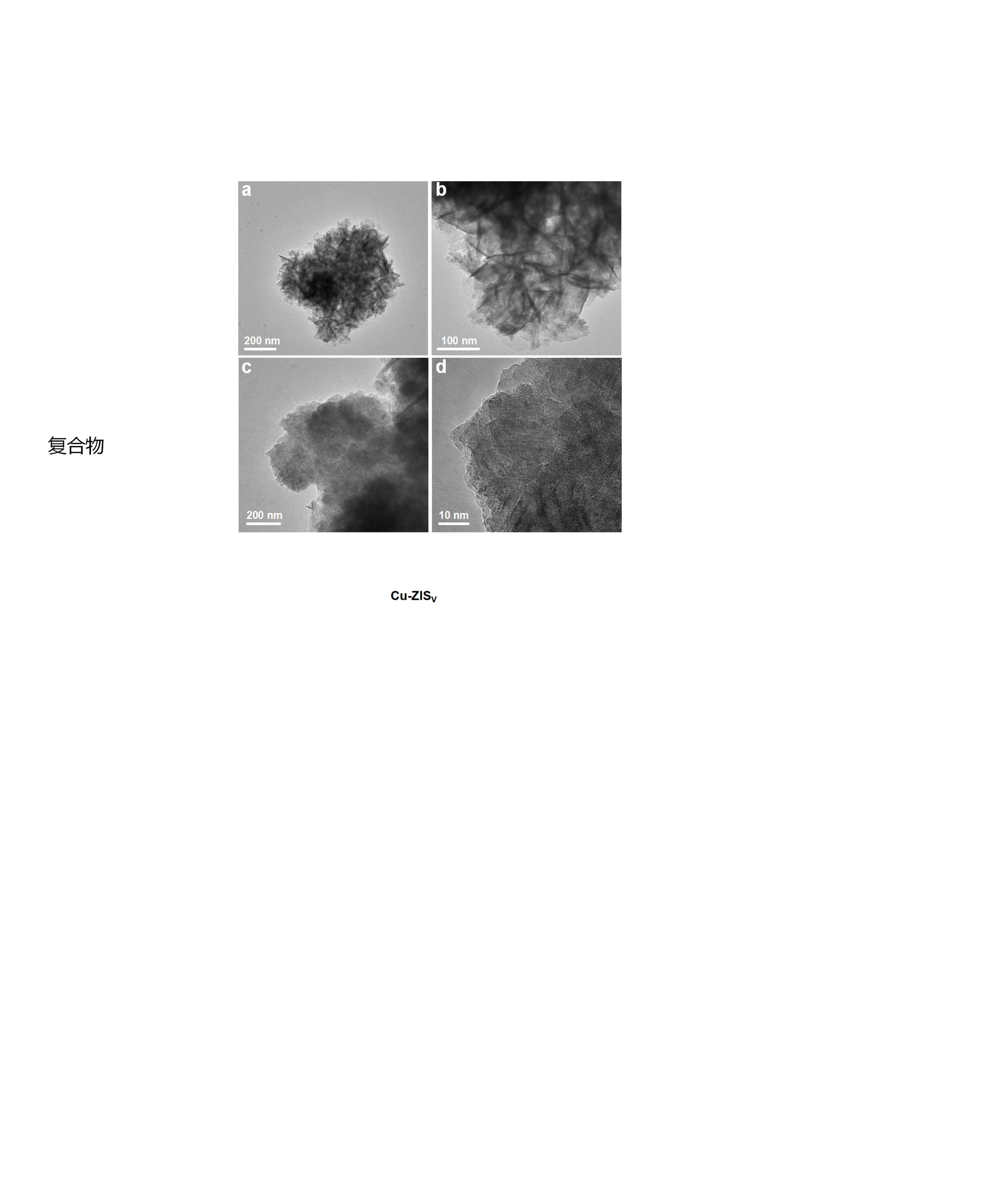
**

**Figure S6.** (a-d) TEM images of Cu-ZIS_V_.

**
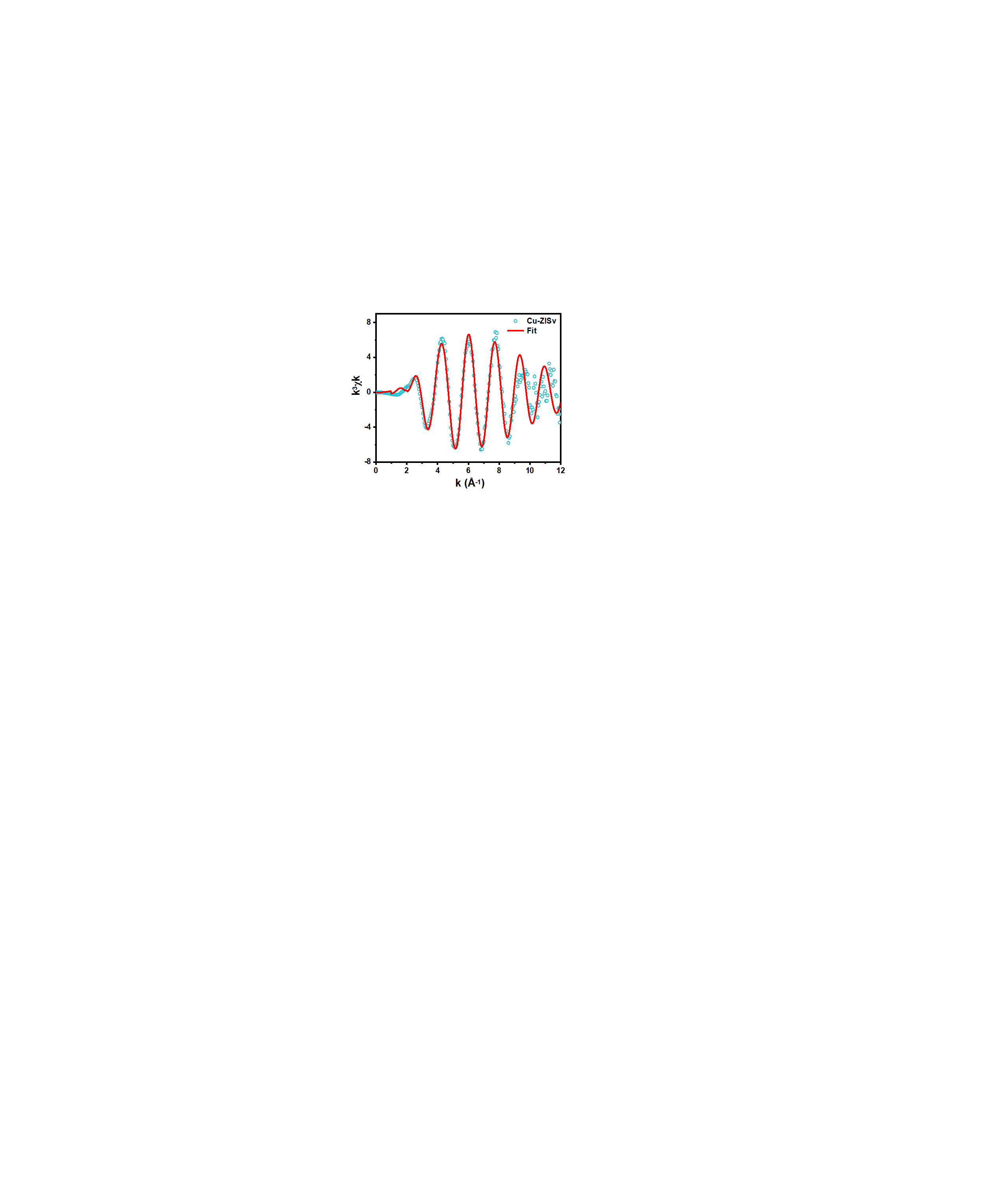
**

**Figure S7.** First-shell fitting of Fourier transformations of EXAFS spectra for Cu-ZIS_V_.

**
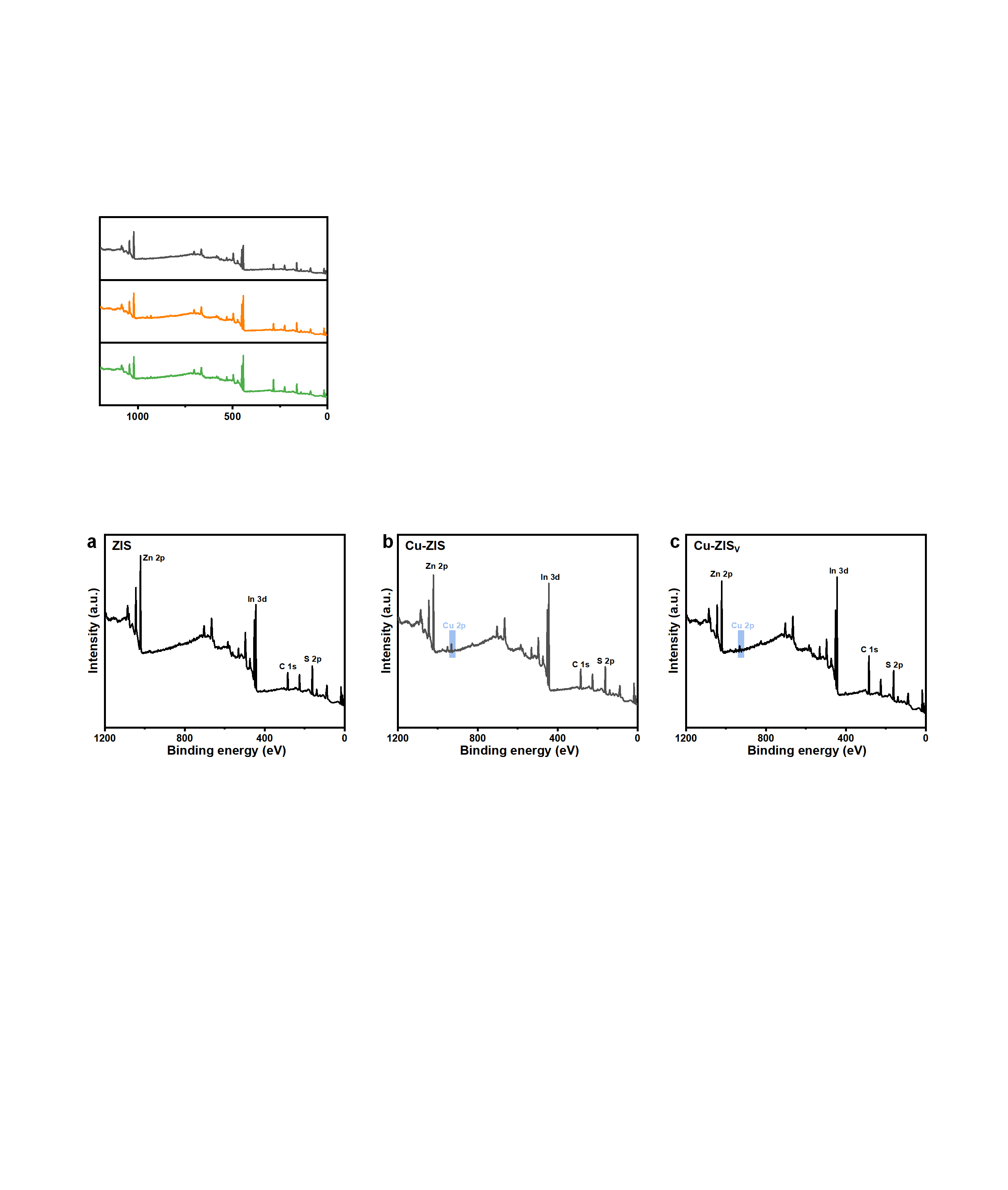
**

**Figure S8.** XPS survey spectra of (a) ZIS, (b) Cu-ZIS, and (c) Cu-ZIS_V_.

**
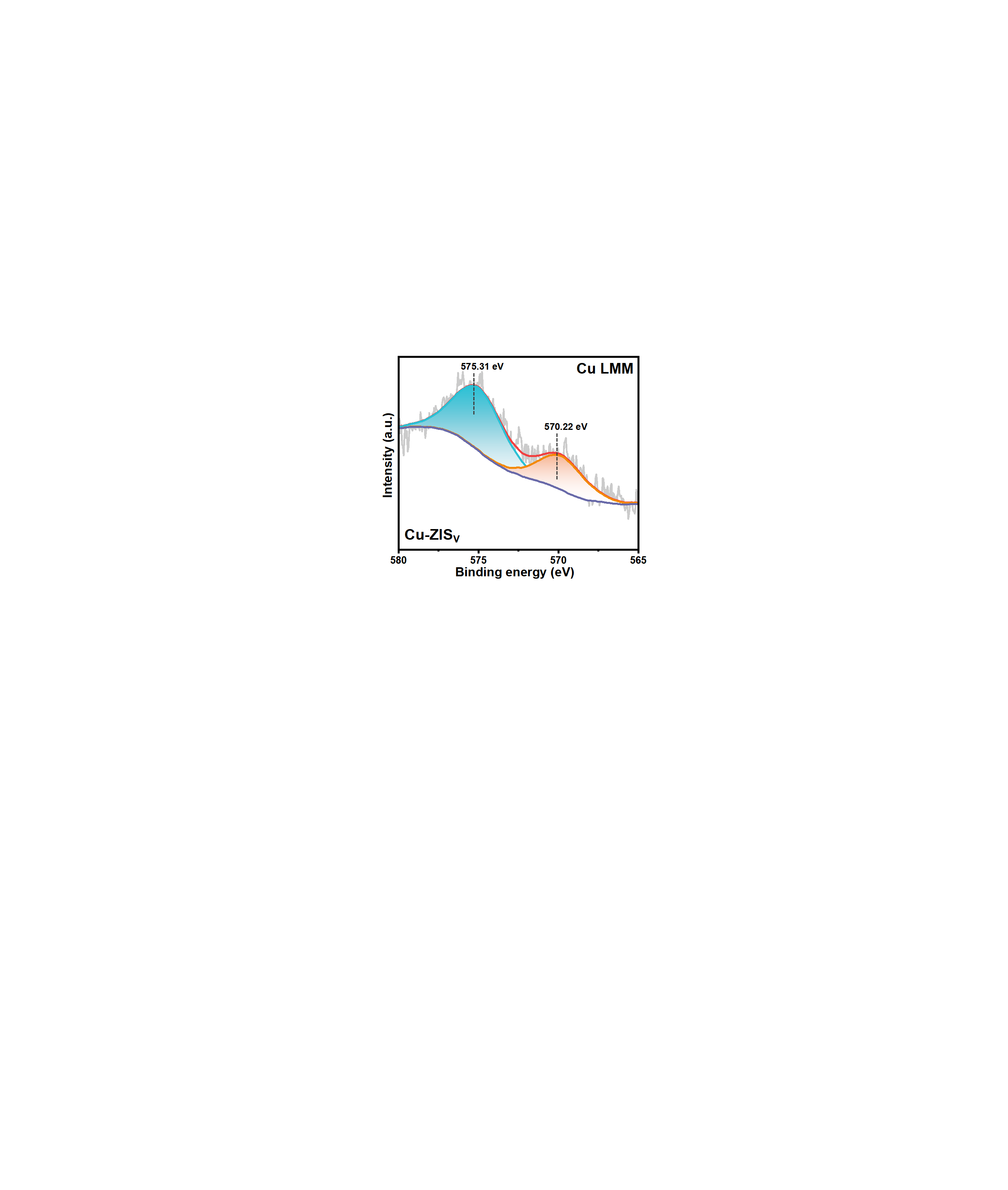
**

**Figure S9.** Cu LMM Auger spectrum of Cu-ZIS_V_.

The auger electron spectroscopy (AES) spectra of Cu-ZIS_V_ confirms the existence of Cu species mainly in the form of Cu^2+^, further validating the close alignment of the Cu K-edge energy with that of copper sulfide.

**
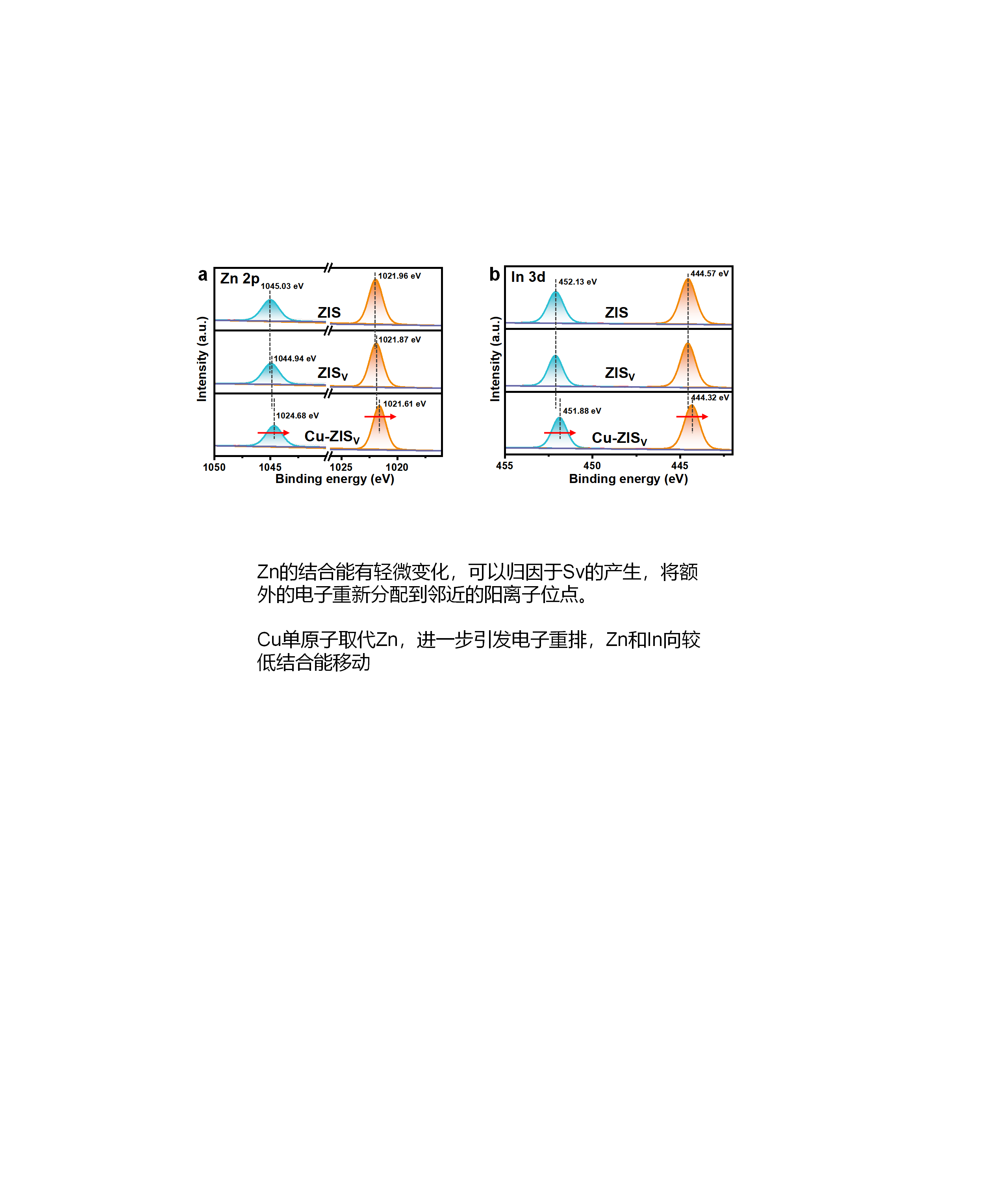
**

**Figure S10.** XPS spectra for the (a) Zn 2p regions and (b) In 3d regions.

The peak positions of Cu-ZIS_V_ in Zn 2p and In 3d XPS spectra are decreased in Figure S10. The Zn 2p_1/2_ and Zn 2p_3/2_ peaks binding energy of Cu-ZIS_V_ (1044.83 and 1021.86 eV) is lower by 0.26 eV compared to ZIS and ZIS_V_ (1045.11 and 1022.14 eV).^32^ For In 3d_3/2_ and In 3d_5/2_ XPS spectra, Cu-ZIS_V_ (452.46 and 444.96 eV) decreased by 0.25 eV over ZIS and ZIS_V_ (452.82 and 445.25 eV). The substitution of Cu atoms for Zn atoms further initiated an electron rearrangement, leading the Zn 2p and In 3d peaks to shift towards a lower binding energy.

**
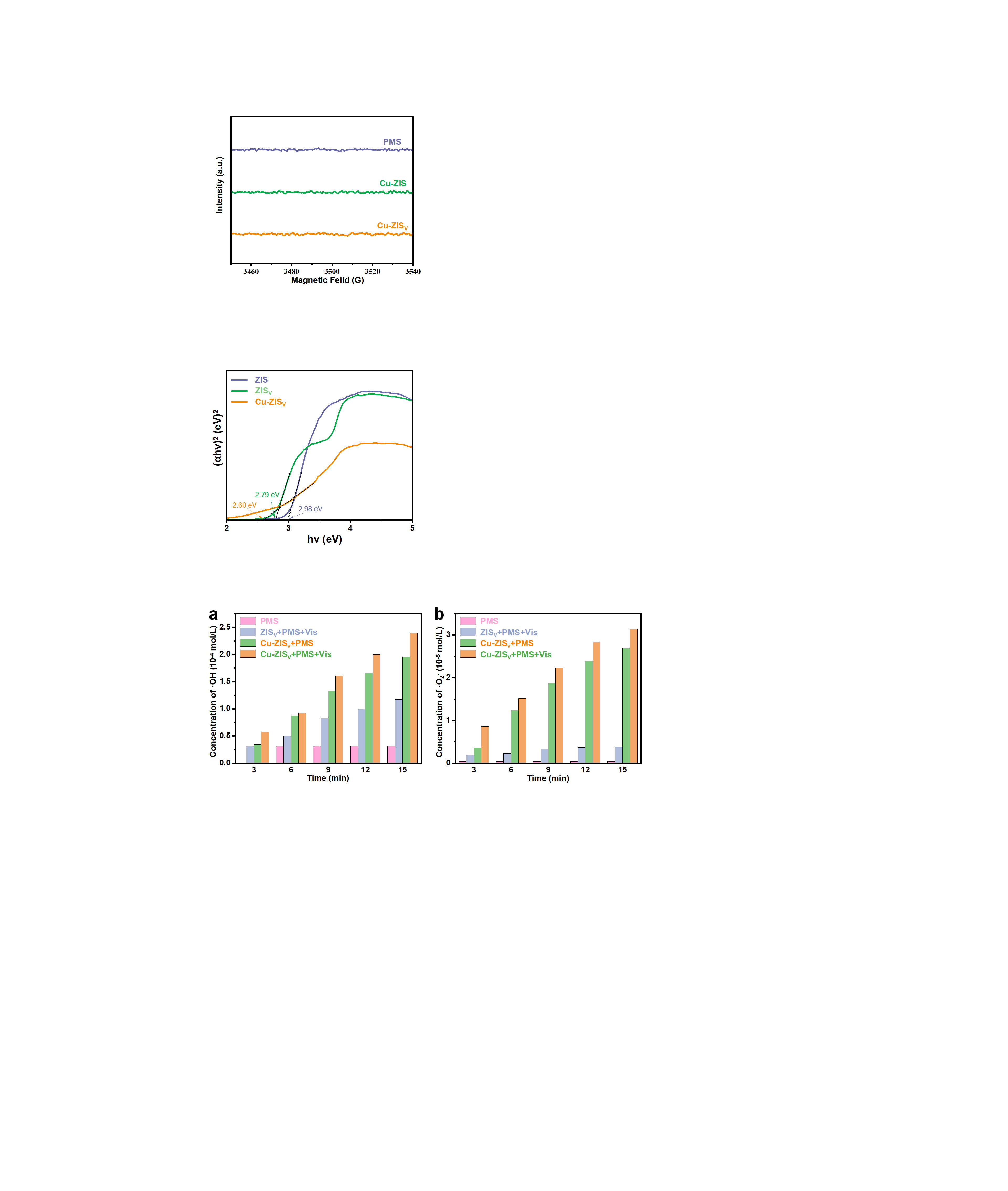
**

**Figure S11.** Band gap of ZIS, ZIS_V_, and Cu-ZIS_V_.

.

**Figure S12.** VB-XPS spectra.

The optical absorption capacities of ZIS, ZIS_V_, and Cu-ZIS_V_ were determined through ultraviolet-visible (UV-vis) diffuse reflection spectroscopy. Figure. 2k shows that the ZIS and ZIS_V_ display light absorption edges at around 475 nm and 505 nm, respectively, while the Cu-ZISv has a light absorption effect throughout the whole visible-light range (380-760 nm). The substitution between Zn atoms and Cu atoms caused by Cu doping improves the visible-light absorption ability of the photocatalyst. The band gap of ZIS, ZIS_V_, and Cu-ZIS_V_ are calculated to be 2.98 eV, 2.79 eV, and 2.60 eV through Kubelka–Munk function, respectively (Figure. S11). As shown in Figure. S12, the valence band (VB) maximum of ZIS, ZIS_V_, and Cu-ZIS_V_ are 1.62 eV, 1.36 eV, and 1.03 eV, respectively. Therefore, the calculated conduction band (CB) potential for ZIS, ZIS_V_, and Cu-ZIS_V_ are approximately −1.36 eV, −1.43 eV, and −1.57 eV, respectively.

**Figure S13.** Transient photocurrent responses of ZIS, ZIS_V_, and Cu-ZIS_V_.

The role of the asymmetric Cu-S_V_-Zn sites in photogenerated electron-hole pairs for efficient migration and transfer is also elucidated by adopting electrochemical impedance spectra (EIS) Nyquist plots and transient photocurrent responses (Figure. 2m, Figure. S13). Compared with ZIS (5.47×10^4^ Ω) and ZIS_V_ (3.88×10^4^ Ω), Cu-ZIS_V_ (1.86×10^4^ Ω) demonstrated the smallest radius of EIS Rt and supported the improved separation and transfer of photogenerated charges in Cu-ZIS_V_. These results indicate that the asymmetric Cu-S_V_-Zn sites induced by self-adapting S vacancies facilitate the efficient separation and transfer of photogenerated charges, which is helpful to the activation of PMS.

**
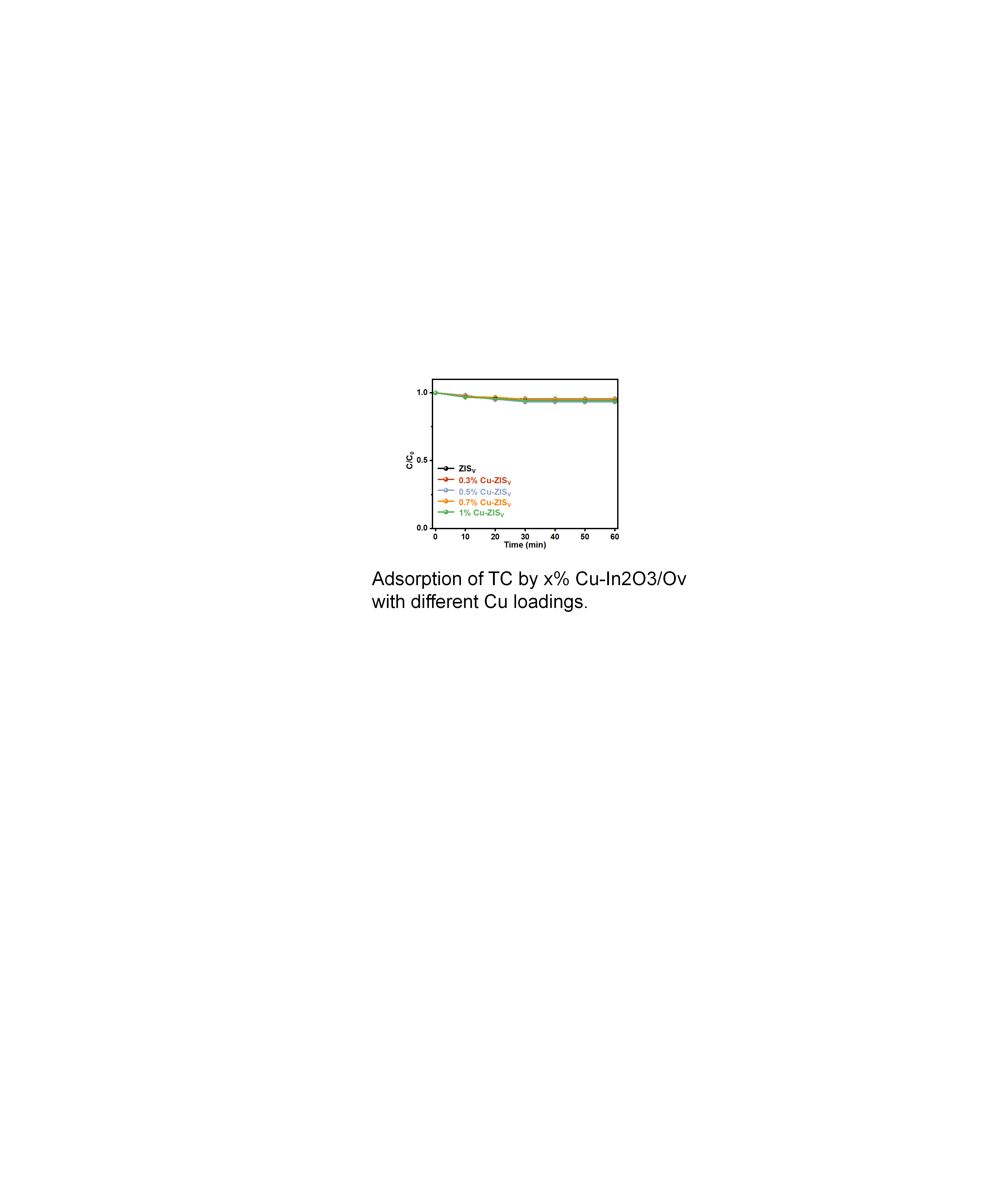
**

**Figure S14.** Adsorption of TC by x% Cu-ZIS_V_ with different Cu loadings.

**
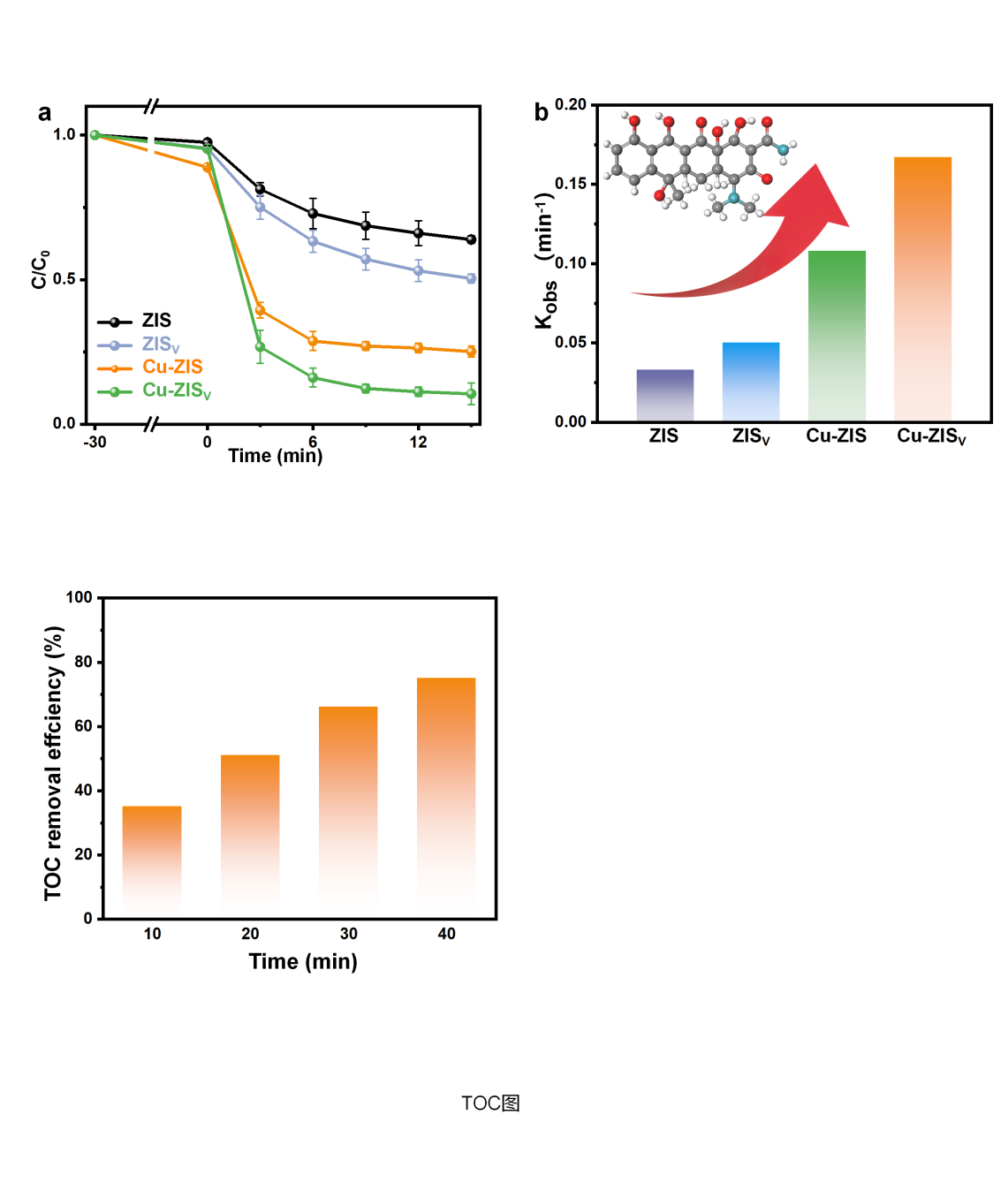
**

**Figure S15.** (a) Degradation efficiency of TC by ZIS, ZIS_V_, Cu-ZIS, and Cu-ZIS_V_. (b) Corresponding first-order rate constants of ZIS, ZIS_V_, Cu-ZIS, and Cu-ZIS_V_.

**
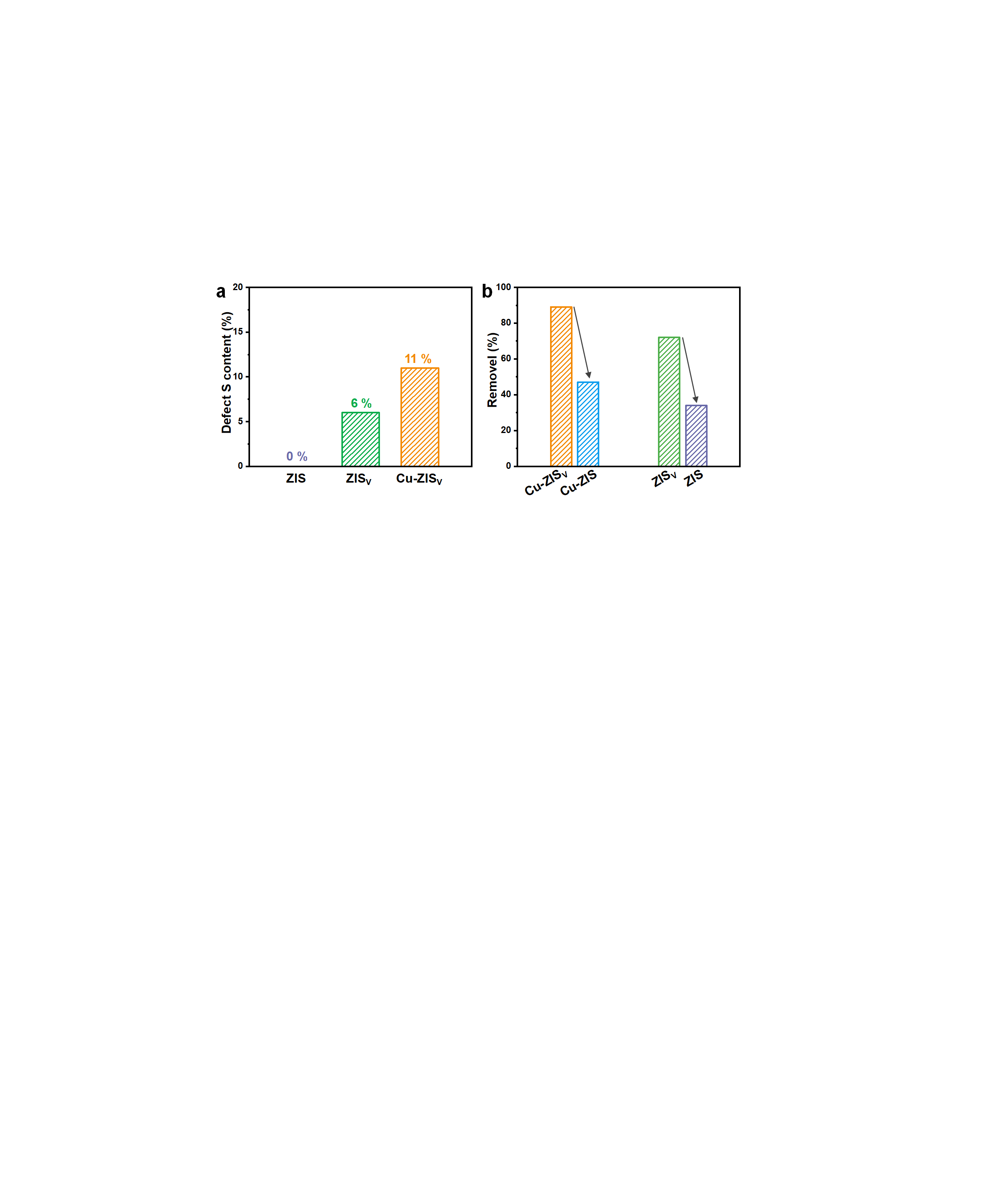
**

**Figure S16.** (a) The relative content of defective S in ZIS, ZIS_V_, and Cu-ZIS_V_ determined by XPS. (b) Comparison of S_V_ degradation performance.

**
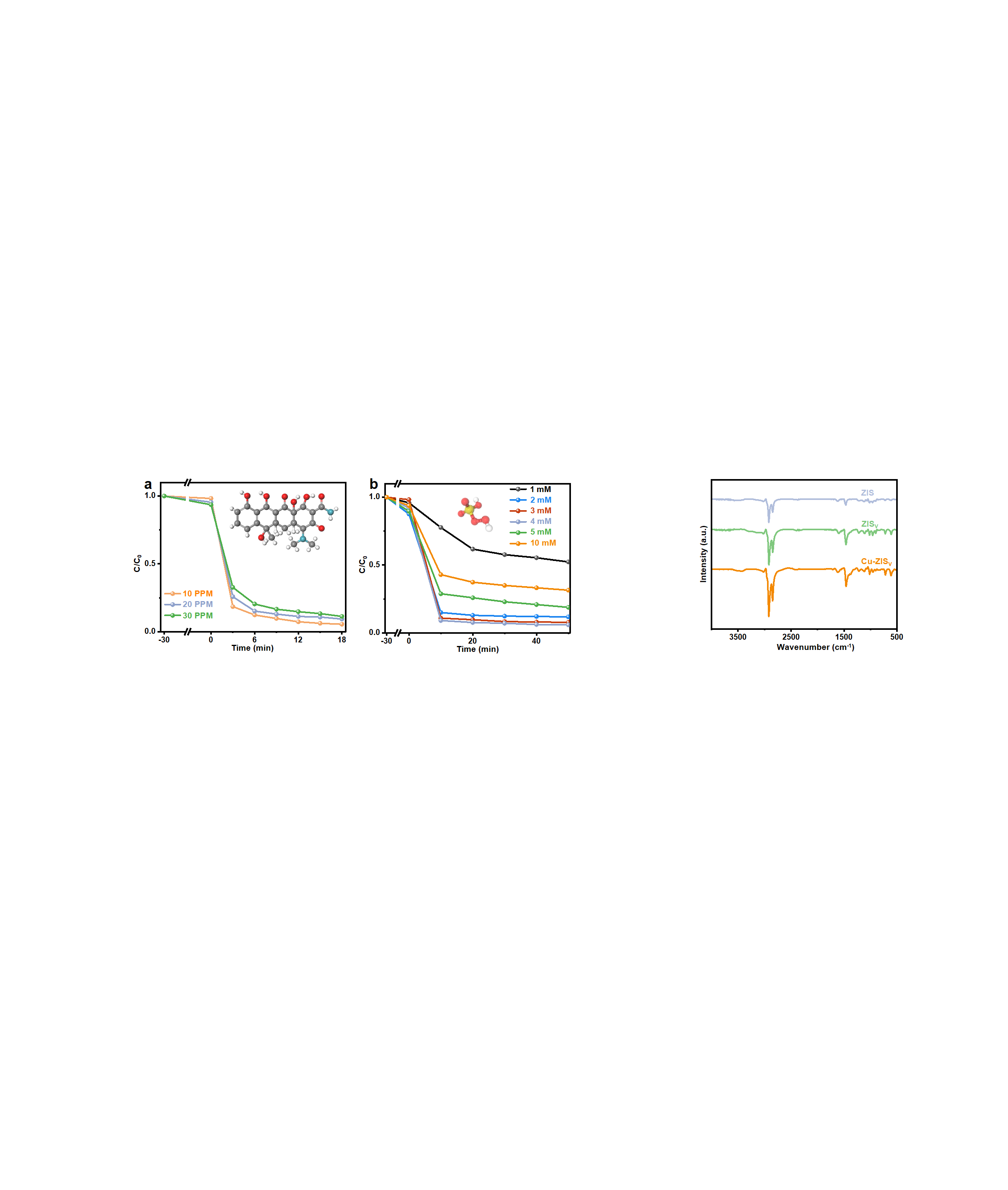
**

**Figure S17.** Degradation of TC by Cu-ZIS_V_ under different conditions: (a) TC concentration, (b) PMS dosage.

As the pollutant concentration increased from 10 PPM to 30 PPM, the Cu-ZIS_V_ exhibited a slightly diminished degradation efficiency and remained above 80%, suggesting effective degradation of TC even at higher concentration levels. Furthermore, the degradation effect of Cu-ZIS_V_ increased with PMS concentration up to 4 mM, beyond which excessive PMS inhibited the performance, likely due to active sites or the formation of lower oxidation potential •SO_5_^-^ form •SO_4_^-^.

**
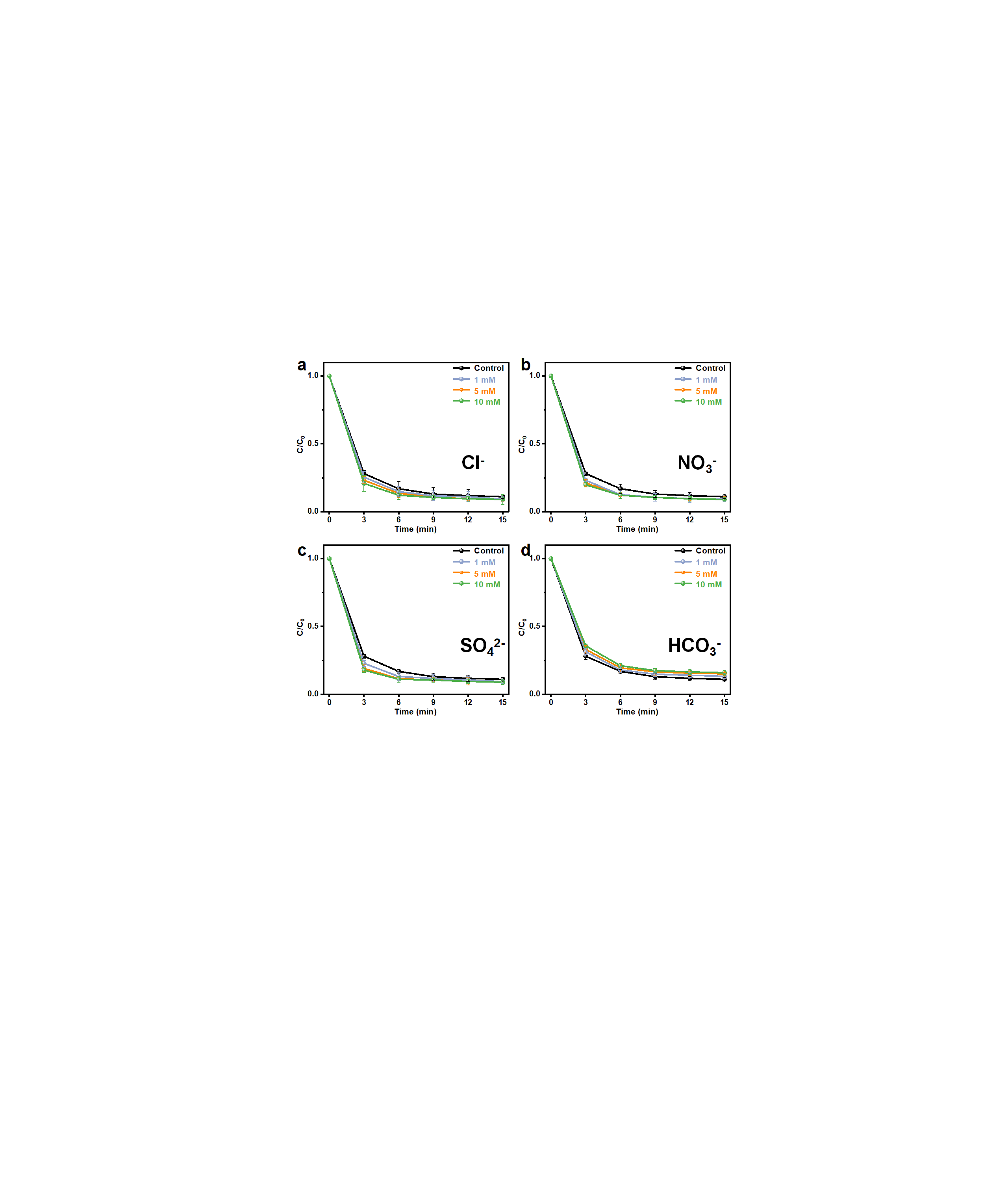
**

**Figure S18.** Effect of (a) Cl^-^, (b) NO_3_^-^, (c) SO_4_^2-^, and (d) HCO_3_^-^ on TC removal by Cu-ZIS_V_.


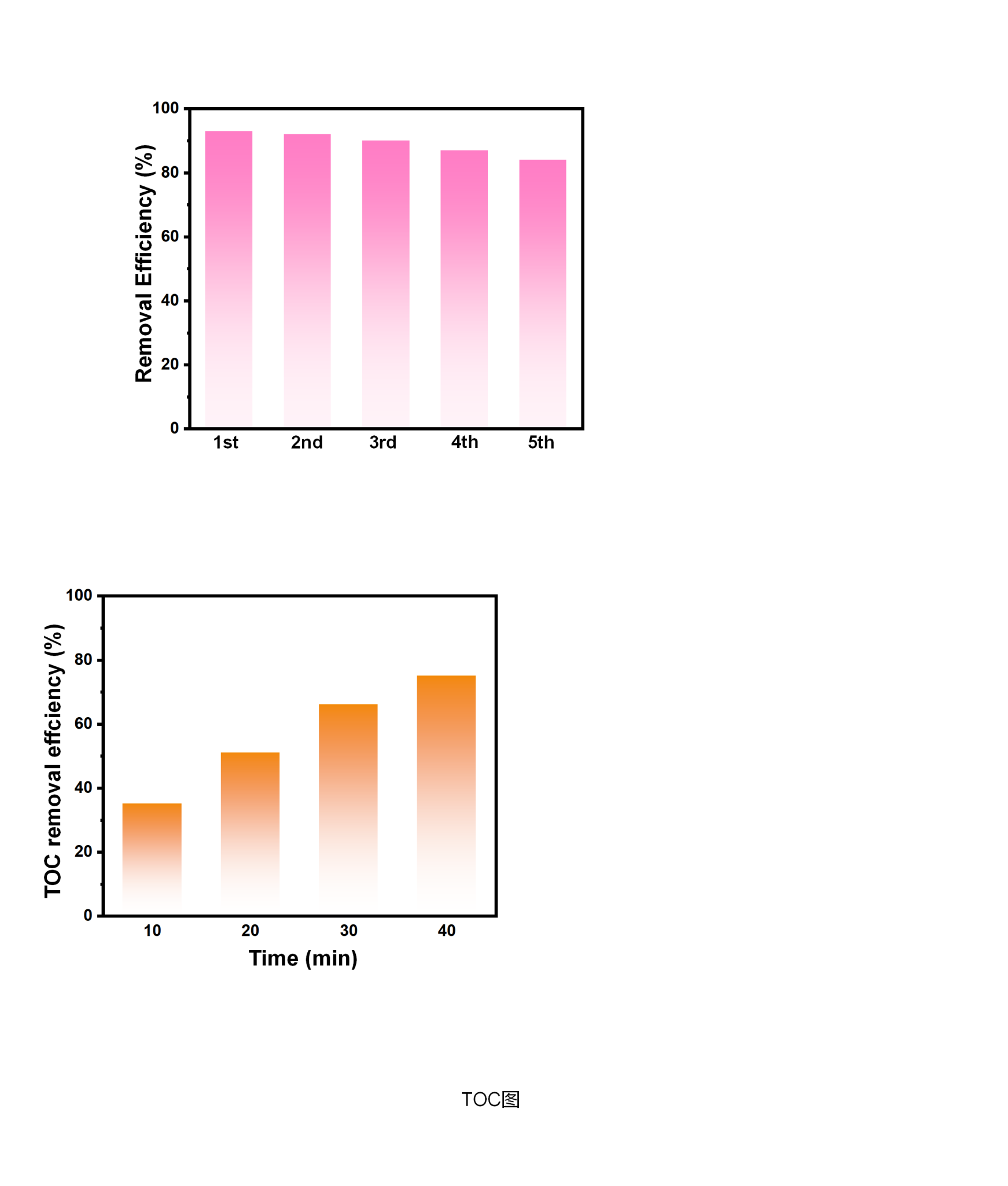


**Figure S19.** The cycling runs in the degradation of TC over Cu-ZIS_V_ + PMS + Vis system.

**
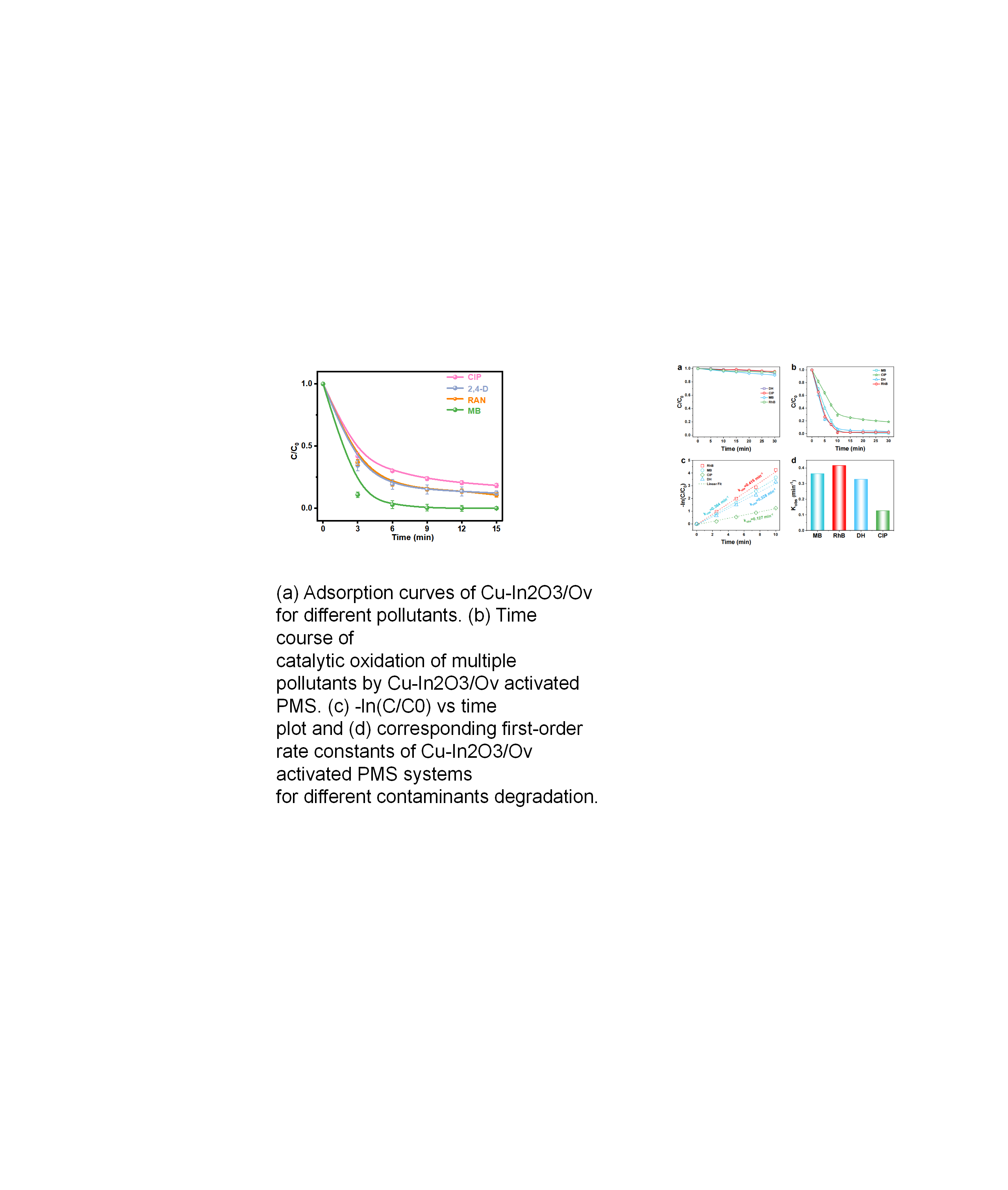
**

**Figure S20.** Time course of catalytic oxidation of multiple pollutants by Cu-ZIS_V_ activated PMS.

**
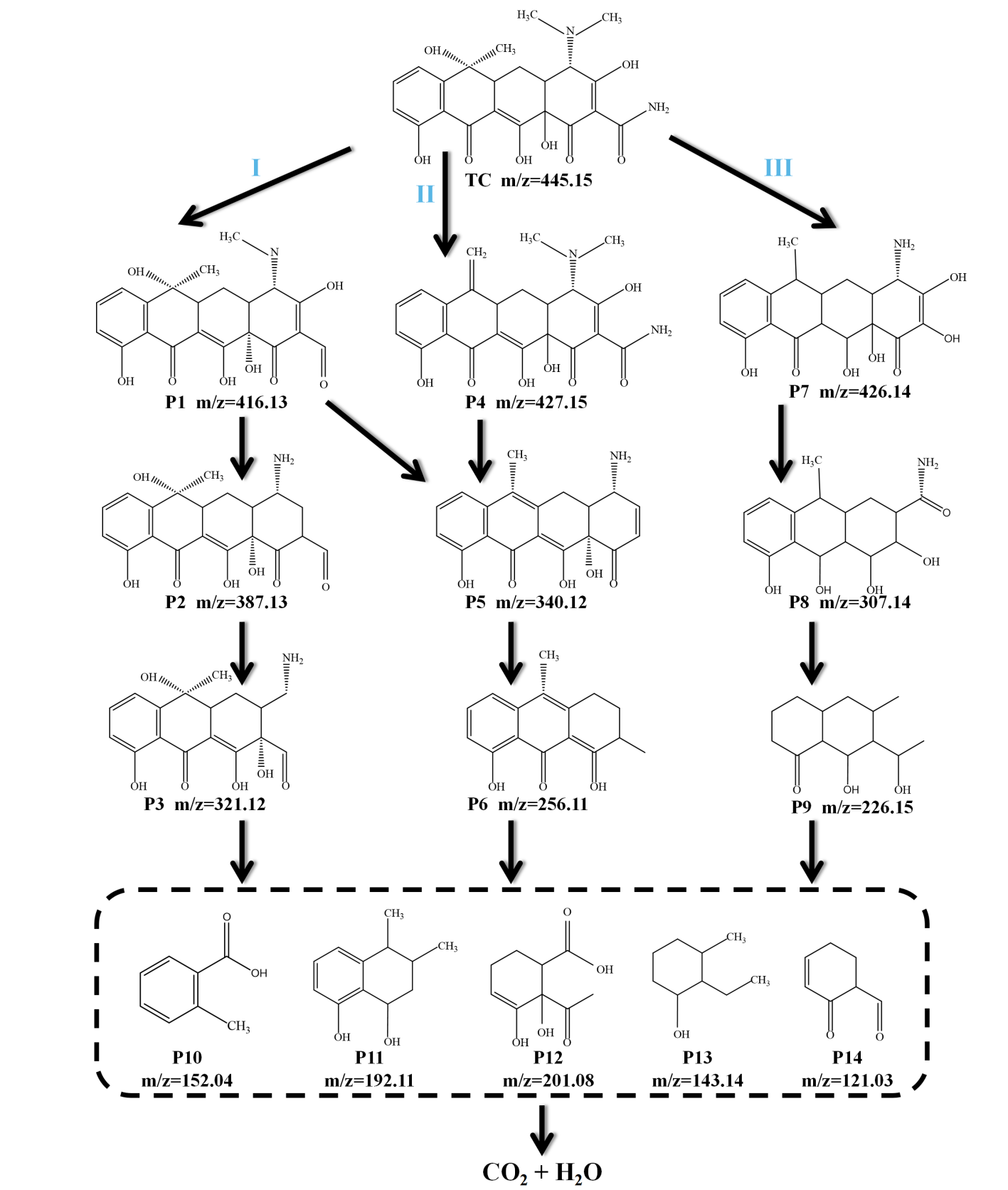
**

**Figure S21.** Proposed degradation pathway of TC in Cu-ZIS_V_ + PMS + Vis system.

Three potential degradation pathways of TC were proposed according to the active species produced during the degradation process. Firstly, TC was attacked by h^+^ with strong oxidation, resulting in the loss of hydroxyl, N-methyl, and -NH, producing P1 (m/z 416.13), P4 (m/z 427.15), and P7 (m/z 426.14), respectively. Through a demethylation process, the P1 was converted to P2 (m/z 387.13). The liberation of nitrogen and the elimination of the amide group during oxidation led to the synthesis of P5 (m/z 340.12). Reaction intermediates underwent hydroxylation, carbosylation, and ring-opening reactions due to further attack by the •OH and •O_2_^-^. Then, the P7 would change to P8 (m/z 307.14). The P3 (m/z 321.12), P6 (m/z 256.11), and P9 (m/z 226.15) were gradually produced as the oxidation reaction continued. The complete cleavage of the broken benzene ring further formed the intermediates P10 (m/z 152.04), P11 (m/z 192.11), P12 (m/z 201.08), P13 (m/z 143.14), and P14 (m/z 121.03). Eventually, the resulting intermediates were carbonated and completely converted into CO_2_ and H_2_O.

**
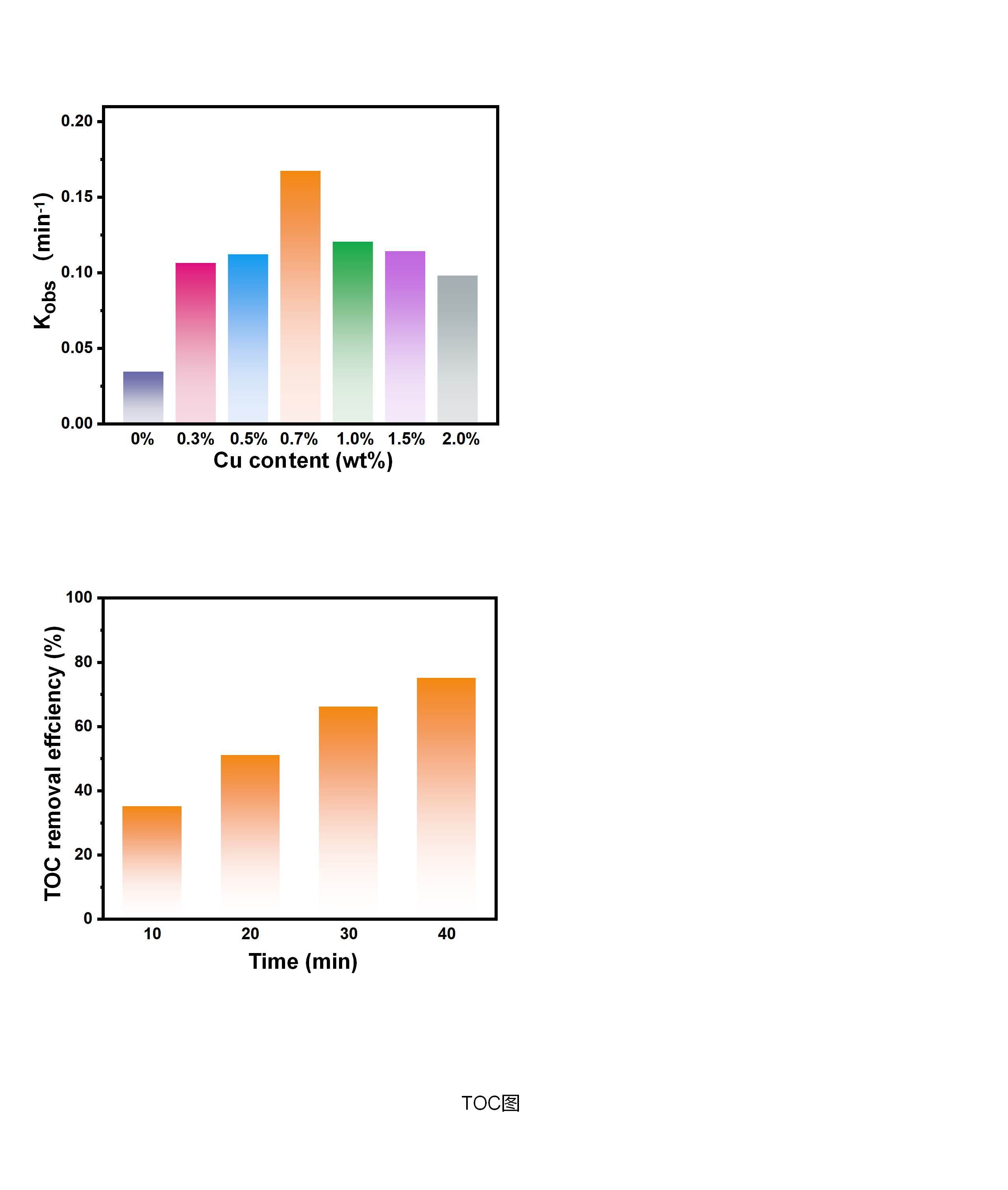
**

**Figure S22.** TOC removal efficiency of TC in Cu-ZIS_V_ + PMS + Vis system.

**
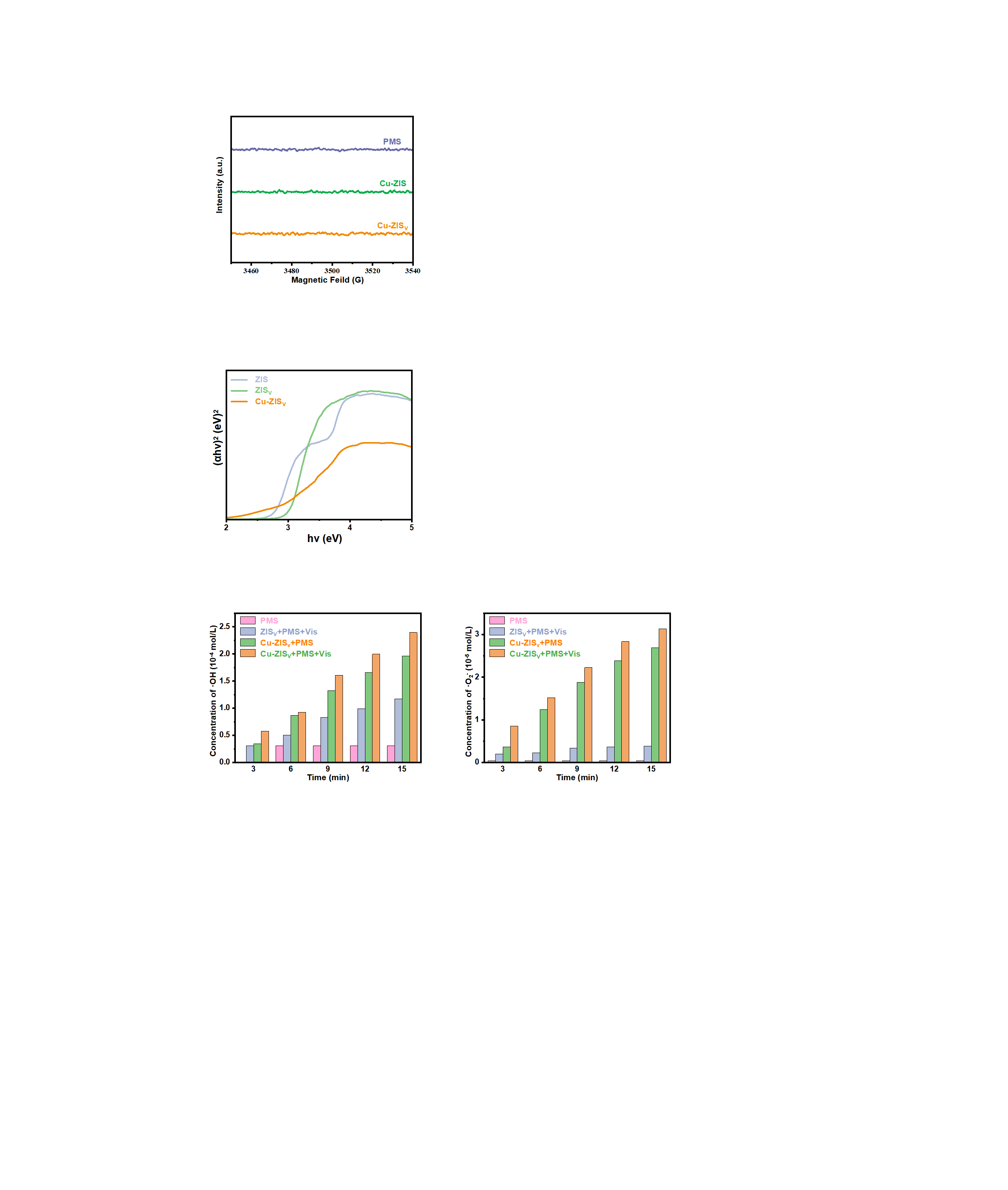
**

**Figure S23.** EPR spectra of ^1^O_2_^-^ using 2,2,6,6-tetramethyl-4-piperidinyl (TEMP) as trapping agent.

**Figure S24.** Retest EPR spectra of ^1^O_2_ in Cu-ZIS_V_ + PMS + Vis system.

**
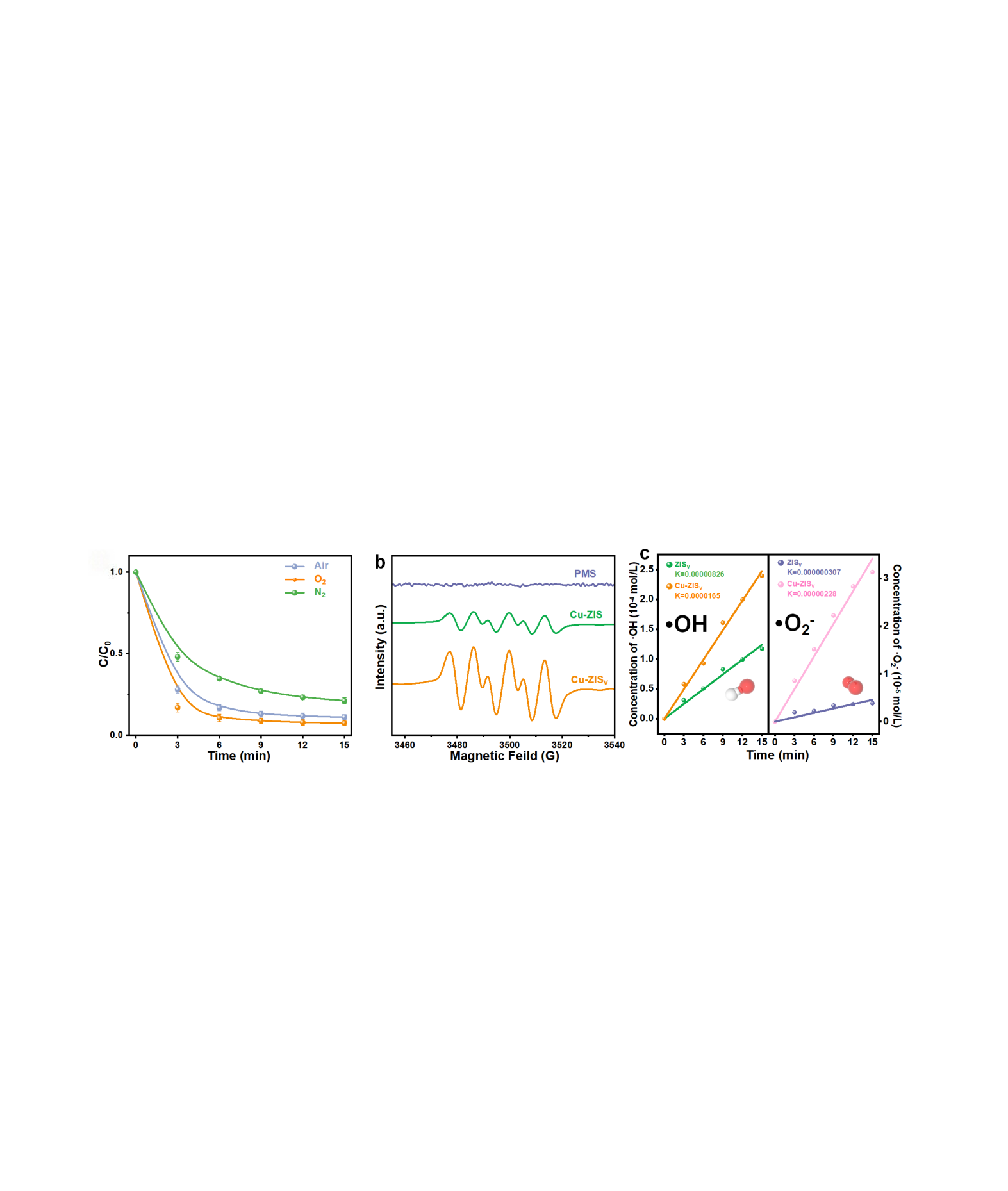
**

**Figure S25.** Degradation efficiency of TC under varying gas conditions.

The molecular oxygen activation performance of Cu-ZIS_V_ was evaluated by conducting degradation TC under different atmospheres (Air, O_2_, and N_2_). Obviously, the presence of O_2_ significantly enhanced the degradation performance of Cu-ZIS_V_, whereas N_2_ had a negative impact on the molecular oxygen activation for Cu-ZIS_V_. This observation indicated that Cu-ZIS_V_ can successfully convert molecular oxygen into ROS, thereby contributing to the efficient oxidation of TC.

**Figure S26.** XPS spectra for the Zn 2p regions of (a) ZIS and (b) ZIS_V_.

**Figure S27.** XPS spectra for the (a) Zn 2p regions and (b) Cu 2p regions of Cu-ZIS_V_.


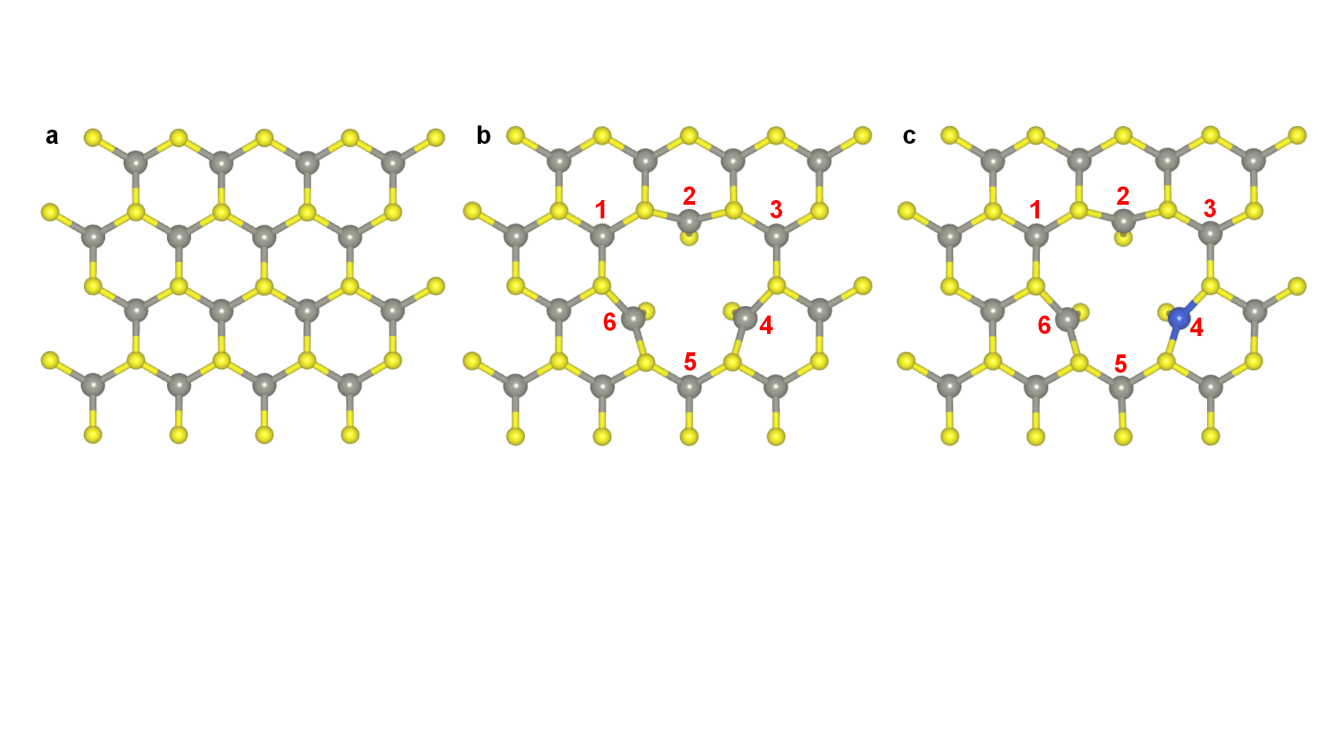


**Figure S28.** The DFT models of ZIS, ZIS_V_, Cu-ZIS_V_.

In density functional theory (DFT) simulations, Cu atom in Z_3_In_2_S_6_ without S vacancies are most stably configured in the Cu-S_4_ structure, where Cu atom lacks free electrons to adsorb PMS due to coordination saturation. In contrast, in Z_3_In_2_S_6_ with S vacancies, Cu atom exists in a Cu-S_3_ structure, and the coordinatively unsaturated state of Cu atom allows for evident interactions with PMS. The S vacancies expose reactive sites on Cu atom, and the unsaturated coordination of Cu atom provides additional free electrons to enhance PMS adsorption and activation.

**Figure S29.** The optimized adsorption configurations of PMS molecules on the surface of ZIS_V_ models (Zn: black; In: purple; O: red; S: yellow; H: white).


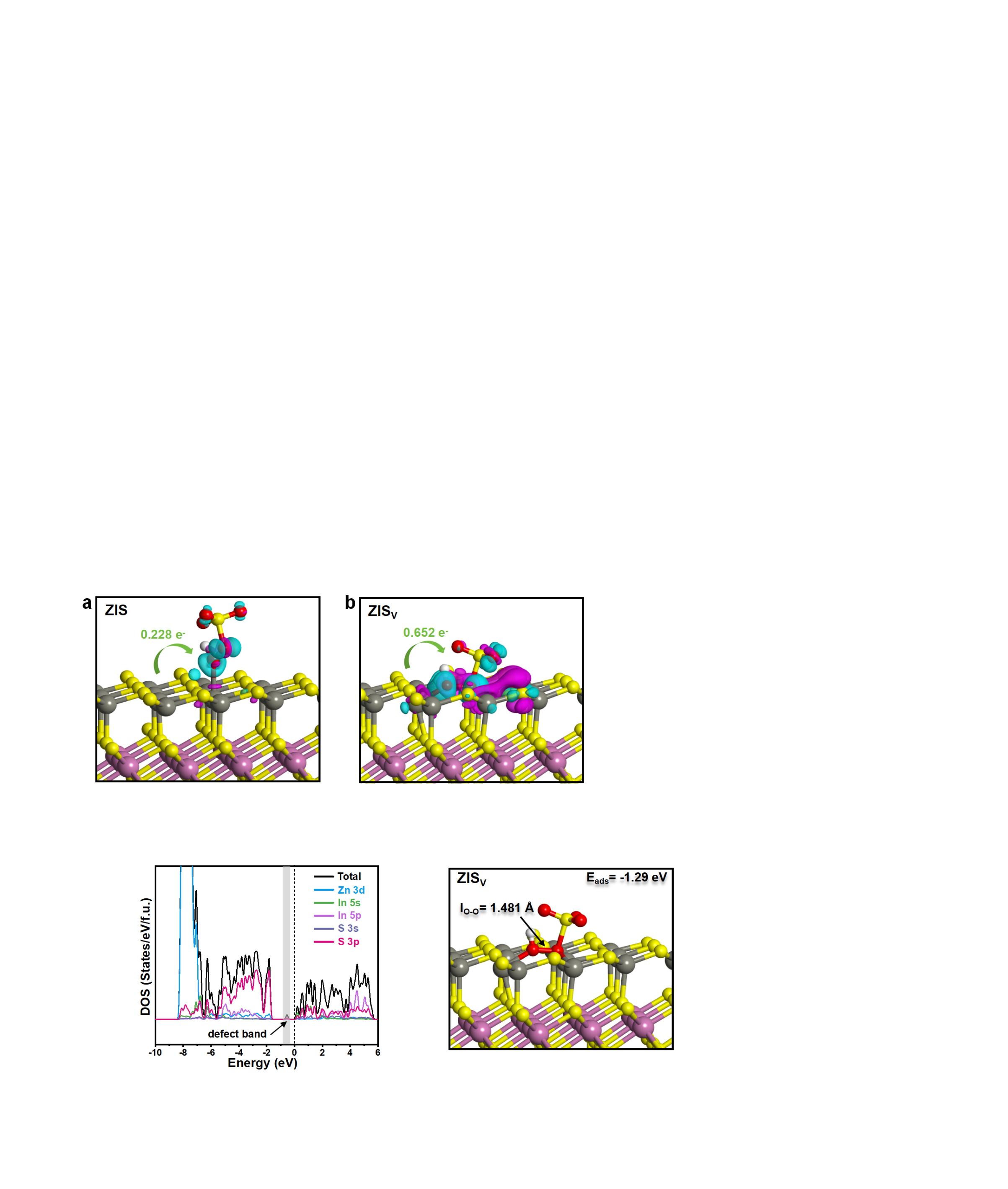


**Figure S30.** Charge density difference and corresponding charge transfer for PMS adsorption on ZIS_V_ were determined using the following equation: Δρ = ρ(total) - ρ(surface) - ρ(PMS).

The blue region represents the electron accumulation, while the purple region represents the electrons depletion.

**Figure S31.** Water flux rate over time of treatment.

**3. Supporting Tables**

**Table S1.** The weight percentage of S, In, Zn and Cu in samples measured by ICP-MS

| Sample | S content  (wt%) | In content  (wt%) | Zn content  (wt%) | Cu |
| --- | --- | --- | --- | --- |
| ZIS | 31.1 | 37.0 | 31.3 |  |
| ZISv | 30.3 | 36.8 | 31.1 |  |
| Cu-ZISv | 28.6 | 36.4 | 27.8 | 6.7 |

The weight percentage of In atom remained almost constant, while the weight percentage of Zn and S decreased with the Cu doped, indicating that the Cu atoms substituted for the Zn atoms.

**Table S2.** EXAFS fitting parameters at the Cu K-edge for Cu-ZISv.

| Sample | Shell | N^a^ | R(Å)^b^ | σ^2^×10^3^(Å^2^)^c^ | ΔE_0_ (eV)d | R factor |
| --- | --- | --- | --- | --- | --- | --- |
| Cu foil | Cu-Cu | 12 | 2.54 | 9.0 | 4.2 | 0.005 |
| CuS | Cu-S | 3.7 | 2.28 | 6.0 | 3.4 | 0.012 |
| Cu_2_S | Cu-S | 3.8 | 2.26 | 8.4 | 1.3 | 0.009 |
| Cu-ZISv | Cu-S | 3.1 | 2.23 | 9.9 | 6.2 | 0.011 |

^a^N: coordination numbers; ^b^R: bond distance; ^c^σ^2^: Debye-Waller factors; ^d^ΔE_0_: the inner potential correction. R factor: goodness of fit.

**Table S3.** The element content (S, In, Zn, Cu) in samples measured by XPS

| Sample | S atomic  (%) | In atomic  (%) | Zn atomic  (%) | Cu |
| --- | --- | --- | --- | --- |
| ZIS | 54.4 | 18.5 | 27.1 |  |
| ZISv | 54.2 | 18.3 | 27.5 |  |
| Cu-ZISv | 51.1 | 18.1 | 24.9 | 5.9 |

**Table S4.** XPS fitting data of area of elements, the atomic ratios of S to In and S vacancies concentrations. The RSF (relative sensitivity factor) for Zn 2p, In 3d, S 2p and Cu 2p are 5.589, 7.265, 0.668 and 5.321 respectively.

| Sample | Element | Area/(T*MFP) | Area/(RSF*T*MFP) | S:In | S vacancies concentrations |
| --- | --- | --- | --- | --- | --- |
| ZIS | Zn 2p | 112015 | 20074.4 | 2.24 | 0 |
|  | In 3d | 86733 | 11930.3 |  |  |
|  | S 2p | 17926 | 26755.2 |  |  |
| ZIS_V_ | Zn 2p | 87287 | 15642.8 | 2.11 | 6% |
|  | In 3d | 92857 | 12772.6 |  |  |
|  | S 2p | 18063 | 26919.4 |  |  |
| Cu-ZIS_V_ | Zn 2p | 68116 | 12207.2 | 2.00 | 11% |
|  | In 3d | 82144 | 11299.0 |  |  |
|  | S 2p | 15187 | 22667.2 |  |  |
|  | Cu 2p | 4861 | 913.6 |  |  |

Equation

$$S:In = \frac{Area (S 2p)}{Area (In 3d)}$$

Area: Area (RSF*T*MFP) of corresponding elements

$$S vacancies concentration = \frac{S:In (ZIS) - S:In (sample)}{S:In (ZIS)}$$

|  |
| --- |

The concentration of In atoms remained essentially unchanged through XPS analysis, thus the quantity of S atoms and In atoms can be evaluated to determine the S vacancy content (Table S3 and S4). The atom ratios of S:In for ZIS, ZIS_V_, and Cu-ZIS_V_ are 2.24, 2.11, and 2.00, and the corresponding S vacancy concentrations are 0%, 5.9%, and 11.2%, respectively, indicating the occurrence of local structural self-adapting S vacancies were induced by Cu-doping.**Table S5** Time-resolved photoluminescence fitting results of ZIS, ZIS_V_ and Cu-ZIS_V_.

| Sample | τ (ns) | A% | τ_ave_ (ns) |
| --- | --- | --- | --- |
| ZIS | τ_1_=0.2635  τ_2_=1.9324  τ_3_=10.5600 | B_1_=42.08  B_2_=34.65  B_3_=23.27 | 3.2373 |
| ZIS_V_ | τ_1_=0.3987  τ_2_=2.1291  τ_3_=15.8433 | B_1_=52.18  B_2_=32.33  B_3_=15.48 | 3.3495 |
| Cu-ZIS_V_ | τ_1_=1.0051  τ_2_=9.1673  τ_3_=73.5142 | B_1_=26.28  B_2_=34.21  B_3_=39.52 | 32.4498 |

Equation

$$\tau_{\mathrm{ave}} = \frac{B_{1}\tau_{1}^{2}+B_{2}\tau_{2}^{2}+B_{3}\tau_{3}^{2}}{B_{1}\tau_{1}+B_{2}\tau_{2}+B_{3}\tau_{3}}$$

$$\kappa_{\mathrm{ET}} = \frac{1}{\tau_{\mathrm{ave}}(ZIS)}-\frac{1}{\tau_{\mathrm{ave}}(\mathrm{ZIS}_{v})} = 0.1035\times{10}^{8}S^{-1}$$

$$\eta_{\mathrm{ET}} = 1-\frac{\tau_{\mathrm{ave}}(ZIS)}{\tau_{\mathrm{ave}}(\mathrm{ZIS}_{v})} = 3.35\%$$

$$\kappa_{\mathrm{ET}} = \frac{1}{\tau_{\mathrm{ave}}(\mathrm{ZIS}_{v})}-\frac{1}{\tau_{\mathrm{ave}}({Cu-ZIS}_{v})} = 0.2677\times{10}^{9}S^{-1}$$

$$\eta_{\mathrm{ET}} = 1-\frac{\tau_{\mathrm{ave}}(\mathrm{ZIS}_{v})}{\tau_{\mathrm{ave}}({Cu-ZIS}_{v})} = 89.68\%$$

where, τ_1_, τ_2_, τ_3_ and τ_ave_ are photogenerated charge lifetime and $\kappa_{\mathrm{ET}}$, $\eta_{\mathrm{ET}}$ are electron transfer rate and electron transfer efficiency, respectively.

The electron transfer rate (κ_ET_) and efficiency (η_ET_) are calculated using the equation provided in Table S5. In ZIS_V_, the κ_ET_ and η_ET_ are found to be 0.1035×10^8^S^-1^ and 3.35%, indicating electron transfer between sulfur vacancies and Zn atoms at Zn-S_V_-Zn sites but apparently constrained by the symmetry structure. However, the κ_ET_ and η_ET_ in Cu-ZIS_V_ have reached 0.2677×10^9^S^-1^ and 89.68%, respectively, demonstrating that the asymmetric Cu-S_V_-Zn sites induced by Cu single atoms allow faster and more efficient electron transfer and offer a favorable environment for the activation of PMS.

**Table S6.** Comparison of the kinetics of pollutant degradation by the state-of-the-art material activating PMS with visible light. The modified kinetic rate constant (k-value) was calculated through dividing the observed rate constant of pollutant by the catalyst dosage and PMS concentration, followed by multiplying pollutant concentration.

| **Catalyst** | **Dosage**  **(g/L)** | **Oxidant (mM)** | **Light**  **source** | **Removal efficiency** | **Pollutant concentration(mg/L)** | **k_obs_ (min^-1^)** | **k-value (min^-1^·M^-1^)** | **Ref.** |
| --- | --- | --- | --- | --- | --- | --- | --- | --- |
| Cu-ZnInS_V_ | 0.4 | 1.5 (PMS) | 300 W  Xenon lamp  (λ > 420 nm) | 93%/15 min | 20 | 0.167 | 5.57 | This work |
| MgIn_2_S_4_/Bi_2_O_3_ | 0.5 | 2.0 (PMS) | 300 W  Xenon lamp  (λ > 420 nm) | 88%/60 min | 20 | 0.029 | 0.58 | [11] |
| Bi_2_O_3_/g-C_3_N_4_ | 0.5 | 2.0 (PMS) | 300 W  Xenon lamp  (λ > 420 nm) | 59%/60 min | 40 | 1.29×10^-3^ | 0.052 | [12] |
| Co-Pd/BiVO_4_ | 0.8 | 0.30 (PMS) | 300 W  Xenon lamp  (λ > 420 nm) | 90%/360 min | 20 | 1.40×10^-2^ | 1.75 | [13] |
| MF | 0.6 | 0.20 (PMS) | 300 W  Xenon lamp  (λ > 420 nm) | 90%/40 min | 10 | 5.10×10^-2^ | 4.25 | [14] |
| Ag@Bi_2_WO_6_/FeWO_4_ | 0.8 | 1.5 | 25 W white-LED-light | 61%/60 min | 10 | 0.020 | 0.167 | [15] |
| CNGO | 0.4 | 0.80 (PMS) | 300 W  Xenon lamp  (λ > 420 nm) | 99%/60 min | 10 | 0.0619 | 1.93 | [16] |
| CTM | 0.3 | 0.10 (PMS) | 300 W  Xenon lamp  (λ > 420 nm) | 99% 45 min | 1.0 | 0.100 | 3.33 | [17] |
| TiO_2_/AB | 0.5 | 3.0 (PDS) | 300 W  Xenon lamp  (λ > 420 nm) | 93%/120 min | 30 | 2.00×10^-2^ | 0.373 | [18] |
| Co-MIL-53(AI) | 0.2 | 0.98 (PMS) | Without light | 94%/120 min | 30 | 3.10×10^-2^ | 4.46 | [19] |
| CuFeO_2_/BC | 0.5 | 50 (H_2_O_2_) | Without light | 88%/300 min | 20 | 6.00×10^-3^ | 4.00×10^-3^ | [20] |
| Fe-CN/BWO | 0.4 | 1.0 (H_2_O_2_) | Without light | 94%/120 min | 10 | 2.30×10^-2^ | 0.550 | [21] |

**Table S7.** Comparison between recently reported photocatalysts with S vancancies in Fenton-like reactions for the degradation of recalcitrant phenolic compounds. The modified kinetic rate constant (k-value) was calculated through dividing the observed rate constant of pollutant by the catalyst dosage and PMS concentration, followed by multiplying pollutant concentration.

| **Catalyst** | **Dosage**  **(g/L)** | **Oxidant (mM)** | **Light**  **source** | **Removal efficiency** | **Pollutant concentration(mg/L)** | **k_obs_ (min^-1^)** | **k-value (min^-1^·M^-1^)** | **Ref.** |
| --- | --- | --- | --- | --- | --- | --- | --- | --- |
| Cu-ZnInS_V_ | 0.4 | 1.5 (PMS) | 300 W  Xenon lamp  (λ > 420 nm) | 93%/15 min | 20 | 0.167 | 5.57 | This work |
| Co_n_-Ni_n_/NCS/SCNC-Sv | 0.5 | 15 (PMS) | 300 W  Xenon lamp  (λ > 420 nm) | 89%/25 min | 100 | 0.082 | 1.09 | [22] |
| SVs-In_2_S_3_/TiO_2_ | 0.36 | 0.5 (PMS) | 300 W  Xenon lamp  (λ > 420 nm) | 92%/60 min | 10 | 0.043 | 2.39 | [23] |
| ZCS2 | 0.8 | 25 (PMS) | 300 W  Xenon lamp  (λ > 420 nm) | 100%/10 min | 10 | 2.303 | 1.15 | [24] |
| 68M-10 | 0.2 | 0.4 (PMS) | 300 W  Without light | 99%/15 min | 10 | 0.115 | 3.59 | [25] |
| 5-MoS | 0.3 | 1.5 | 300 W tungsten lamp | 83.6%/30 min | 20 | 0.085 | 3.78 | [26] |
| FeS_2_@Mo_2_C | 0.133 | 0.333 (PMS) | 300 W  Xenon lamp  (λ > 420 nm) | 100%/35 min | 0.03 | 0.085 | 0.058 | [27] |
| SK-Co(1.0) | 0.3 | 12 (PMS) | Without light | 91.56% 30 min | 10 | 0.187 | 0.519 | [28] |
| Zr-Co_x_S_y_-V | 15 | 0.3 (PMS) | Without light | 100%/15 min | 4 | 0.332 | 0.295 | [29] |
| nZVI@MoS | 0.1 | 0.8 (PDS) | Without light | 97.8%/30 min | 0.02 | 0.124 | 0.013 | [30] |

**Table S8.** The main compositions of the real water samples.

| **Parameters** | **Tap water** | **Lake water** | **Waste water** |
| --- | --- | --- | --- |
| TOC (mg L^-1^) | 1.28 | 6.89 | 473 |
| DO (mg L^-1^) | 5.77 | 7.46 | 5.89 |
| pH (25℃) | 7.31 | 7.11 | 6.68 |
| Cl^-^ (mg L^-1^) | 3.79 | 12.49 | 376 |
| NO_3_^-^ (mg L^-1^) | 2.69 | 9.02 | 4.68 |
| SO_4_^2-^ (mg L^-1^) | 21.3 | 56.68 | 120.58 |

Tap water sampled from Haihe Education Park (Tianjin, China), lake water sampled from Mati Lake (Nankai University, China), and waste water sampled from Jinnan Wastewater Treatment Plant (Tianjin, China).

As shown in Table S8, the residual organic matter (473 mg L^-1^ of TOC) and chloride (376 mg L^-1^ of Cl^-^) in wastewater were the main reasons that led to the decline of TC degradation efficiency: (i) the residual organic matter competed with TC for the •OH/•SO_4_^-^ generated by PMS activation, and (ii) the Cl^-^ converted the •OH/•SO₄⁻ into less reactive secondary radicals or ionic intermediates, thereby reducing the effective concentration and oxidative capacity free radicals in photo-Fenton-like system. In conclusion, the combined effects of residual organic matter and inorganic anions were the main reasons contributing to the reduced TC degradation efficiency (from 93% to 76%) in wastewater.

**Table S9.** Products of TC degradation in the Cu-ZIS_V_ + PMS + Vis system.

| Compounds | Formula | m/z | Structure |
| --- | --- | --- | --- |
| TC | C_22_H_24_N_2_O_8_ | 455.15 |  |
| P1 | C_21_H_21_NO_8_ | 416.13 |  |
| P2 | C_20_H_21_NO_7_ | 387.13 |  |
| P3 | C_16_H_19_NO_6_ | 321.12 |  |
| P4 | C_22_H_20_N_2_O_7_ | 427.15 |  |
| P5 | C_19_H_17_NO_5_ | 340.12 |  |
| P6 | C_16_H_15_O_3_ | 256.11 |  |
| P7 | C_22_H_22_N_2_O_7_ | 426.14 |  |
| P8 | C_16_H_21_NO_5_ | 307.14 |  |
| P9 | C_13_H_22_O_3_ | 226.15 |  |
| P10 | C_8_H_8_O_3_ | 152.04 |  |
| P11 | C_14_H_16_O_3_ | 192.11 |  |
| P12 | C_9_H_12_O_5_ | 201.08 |  |
| P13 | C_7_H_6_O | 143.14 |  |
| P14 | C_7_H_5_O_2_ | 121.03 |  |

**Table S10.** Toxic values of TC and its TPs obtained from T.E.S.T. program. (the Fathead minnow LC_50_ (96 hr), Oral rat LD_50_, Daphnia magna LC_50_ (48 hr), developmental toxicity, Bioaccumulation factor, and mutagenicity were calculated by Toxicity Estimation Software Tool (T.E.S.T) based on Quantitative Structure-Activity Relationship (QSAR)).

| Compounds | Bioaccumulation  factor | Developmental  toxicity | Mutagenicity | Fathead minnow LC_50_  (96 hr) mg/L | Daphnia magna  LC_50_  (48 hr) mg/L | Oral rat  LD_50_  mg/kg |
| --- | --- | --- | --- | --- | --- | --- |
| TC | 0.71 | 0.86 | 0.60 | 0.90 | 8.73 | 806.96 |
| P1 | N/A | 0.82 | 0.66 | 0.76 | 9.78 | 2011.96 |
| P2 | N/A | 0.84 | 0.80 | 3.08 | 8.04 | 5192.83 |
| P3 | N/A | 0.62 | 0.81 | 8.0 | 6.45 | N/A |
| P4 | 1.55 | 0.92 | 0.57 | 0.29 | 1.99 | 1360.66 |
| P5 | 5.25 | 0.88 | 0.67 | 0.33 | 3.12 | 1900.81 |
| P6 | 20.37 | 0.83 | 0.35 | 0.31 | 2.33 | 68.08 |
| P7 | 1.00 | 0.76 | 0.82 | 1.22 | 6.98 | 1347.96 |
| P8 | 1.33 | 0.89 | 0.84 | 6.63 | 27.23 | 978.83 |
| P9 | 7.89 | 0.75 | 0.11 | 26.66 | 94.83 | 255.12 |
| P10 | 1.14 | 0.33 | 0.04 | 68.13 | 60.61 | N/A |
| P11 | 8.95 | 0.94 | 0.45 | 23.2 | 36.5 | N/A |
| P12 | 0.23 | 0.61 | -0.04 | 923.88 | 702.66 | N/A |
| P13 | 3.57 | 0.66 | 0.52 | 28.5 | 87.7 | 2971.41 |
| P14 | N/A | 0.25 | 0.34 | 28.36 | 83.27 | N/A |

**Table S11.** Contribution rates of various active species on tetracycline degradation in Cu-ZIS_V_ +PMS +Vis system

| **Active species** | **Steady-state concentration (M)** | **Second-order reaction rate constants (M^-1^ s^-1^)** | **Reference** | **Contribution value (s^-1^)** | **Contribution rate (%)** |
| --- | --- | --- | --- | --- | --- |
| •OH | 1.0 × 10^-4^ | 4.6 × 10^9^ | [31] | 4.6 × 10^5^ | 82.4 |
| •SO_4_^-^ | 4.1 × 10^-5^ | 2.2 × 10^9^ | [31] | 9.0 × 10^4^ | 16.1 |
| •O_2_^-^ | 2.1 × 10^-5^ | 3.7 × 10^8^ | [32,33] | 7.8 × 10^3^ | 1.5 |

*C*: Steady-state concentration; *k*: Second-order reaction rate constants; *P*: Contribution value; *R*: Contribution rate.

Equation

$$\text{P}\text{(}\text{•OH}\text{)}\text{ =}\text{ }\text{C}\text{(}\text{•OH}\text{)×}\text{k}\text{(}\text{•OH}\text{)}$$

$$\text{P}\text{(}\text{•SO}_{\text{4}}^{\text{−}}\text{)}\text{ =}\text{ }\text{C}\text{(}\text{•SO}_{\text{4}}^{\text{−}}\text{)×}\text{k}\text{(}\text{•SO}_{\text{4}}^{\text{−}}\text{)}$$

$$\text{P}\text{(}\text{•O}_{\text{2}}^{\text{−}}\text{)}\text{ =}\text{ }\text{C}\text{(}\text{•O}_{\text{2}}^{\text{−}}\text{)×}\text{k}\text{(}\text{•O}_{\text{2}}^{\text{−}}\text{)}$$

$$\text{P}\text{(}\text{total}\text{)}\text{ =}\text{ }\text{P}\text{(}\text{•OH}\text{)}\text{+}\text{P}\text{(}\text{•SO}_{\text{4}}^{\text{−}}\text{)+}\text{P}\text{(}\text{•O}_{\text{2}}^{\text{−}}\text{)}$$

$$\text{R}\text{(}\text{•OH}\text{)}\text{ =}\text{ (}\frac{\text{P}\text{(}\text{•OH}\text{)}}{\text{P}\text{(}\text{total}\text{)}}\text{)×100\%}$$

$$\text{R}\text{(}\text{•SO}_{\text{4}}^{\text{−}}\text{)}\text{ =}\text{ (}\frac{\text{P}\text{(}\text{•SO}_{\text{4}}^{\text{−}}\text{)}}{\text{P}\text{(}\text{total}\text{)}}\text{)×100\%}$$

$$\text{R}\text{(}\text{•O}_{\text{2}}^{\text{−}}\text{)}\text{ =}\text{ (}\frac{\text{P}\text{(}\text{•O}_{\text{2}}^{\text{−}}\text{)}}{\text{P}\text{(}\text{total}\text{)}}\text{)×100\%}$$

**Table S12** The calculated adsorption energy of PMS on (001) surfaces of ZIS, ZIS_V_ and Cu-ZIS_V_ (unit: eV).

| Sample | E(total) | E(surface) | E(PMS) | ΔE(ads) | I(O-O) |
| --- | --- | --- | --- | --- | --- |
| ZIS | -456.42109107 | -419.35529945 | -36.49287186 | -0.57291976 | 1.362 Å |
| ZIS_V_ | -453.39189171 | -415.60317434 | -36.49287186 | -1.29584551 | 1.481 Å |
| Cu-ZIS_V_ | -454.29534526 | -416.36837521 | -36.49287186 | -1.43409819 | 1.493 Å |

**Table S13.** The elongation of O–O, S–O, and O–H bonds in PMS with different catalysts.

| **Samples** | **O–O** | **S**–**O (peroxide side)** | **O**–**H** |
| --- | --- | --- | --- |
| PMS | 1.354 | 1.925 | 1.043 |
| ZIS | 1.362 | 1.859 | 1.073 |
| ZIS_V_ | 1.481 | 1.984 | 1.018 |
| Cu-ZIS_V_ | 1.493 | 1.863 | 1.019 |

**Table S14.** The calculated charge transfer of PMS on (001) surfaces of ZIS_V_ and Cu-ZIS_V_ (unit: eV).

|  | Q(surface) | Q(PMS) | ΔQ(surface🡪PMS) |
| --- | --- | --- | --- |
| ZIS | +0.228 | -0.228 | 0.228 |
| ZIS_V_ | +0.652 | -0.652 | 0.652 |
| Cu-ZIS_V_ | +0.727 | -0.727 | 0.727 |

**Table S15** The calculated intermediate energies on (001) surfaces of ZIS_V_ and Cu-ZIS_V_, unit: eV.

| Sample | Energies | surface+PMS | PMS* | OH*+SO_4_* | *+•OH + •SO_4_^-^ |
| --- | --- | --- | --- | --- | --- |
| ZIS | E(ZIS) | -455.84817131 | -456.42109107 | -457.59721433 | -453.79172568 |
|  | ΔE | 0.00000000 | -0.57291976 | -1.17612326 | 3.80548865 |
|  | plot energy | 0.00000000 | -0.57291976 | -1.74904302 | 2.05644563 |
| ZIS_V_ | E(ZIS_V_) | -452.09604620 | -453.39189171 | -454.03382566 | -450.03960057 |
|  | ΔE | 0.00000000 | -1.29584551 | -0.64193395 | 3.99422509 |
|  | plot energy | 0.00000000 | -1.29584551 | -1.93777946 | 2.05644563 |
| Cu-ZIS_V_ | E(Cu-ZIS_V_) | -452.86124707 | -454.29534526 | -454.47045243 | -450.80480144 |
|  | ΔE | 0.00000000 | -1.43409819 | -0.17510717 | 3.66565099 |
|  | plot energy | 0.00000000 | -1.43409819 | -1.60920536 | 2.05644563 |

S vacancy can be expressed as follows:

Step1 (Surface 🡪 Adsorption): * + PMS 🡪 end-on PMS*/side-on PMS*

Step2 (Adsorption 🡪 Dissociation): end-on PMS*/side-on PMS* 🡪 OH* + SO_4_*

Step3 (Dissociation 🡪 Desorption): OH* + SO_4_* 🡪 * + •OH + •SO_4_^-^

where * presents the (001) surfaces of ZIS_V_ and Cu-ZIS_V_ with S vacancy, and intermediates* denotes the corresponding absorbed intermediates. Since the formation of OH* and SO_4_* is an exothermic reaction, the adsorption and dissociation processes of PMS activation occur spontaneously.

**4. References**

[1] G. Kresse, J. Furthmüller, *Comput. Mater. Sci.*, ****1996****, *6*, 15–50.

[2] J. P. Perdew, K. Burke, M. Ernzerhof, *Phys. Rev. Lett.*, ****1996****, *77*, 3865.

[3] P. E. Blöchl, *Phys. Rev. B.*, ****1994****, *50*, 17953.

[4] G. Kresse, D. Joubert, *Phys. Rev. B.*, ****1999****, *59*, 1758.

[5] S. Grimme, *J. Comput. Chem.*, ****2006****, *27*, 1787–1799.

[6] S. Grimme, J. Antony, S. Ehrlich, H. Krieg, *J. Chem. Phys.*, ****2010****, *132*, 154104.

[7] M. Yuan, J. Suriyaprakash, L. Shan, H. Xu, X. Li, H. Wu, G. Ding, Z. Shi, L. Dong, F. M. Zhang, J. Colloid Interface Sci. **2024**, 658, 571–583.

[8] M. Garcia-Ratés, N. López, J. Chem. Theory Comput. **2016**, 12, 1331–1341.

[9] D. Zhang, H. Li, J. Mater. Chem. A **2024**, 12, 13742–13750.

[10] D. Vassetti, I. C. Oǧuz, F. Labat, J. Chem. Theory Comput. **2021**, 17, 6432–6448.

[11] C. Zhang, J. Ni, N. Ding, H. Liu, *Catal. Commun.*, ****2023****, *183*, 106773.

[12] M. Long, D. Li, Q. Zhao, H. Li, Q. Wen, L. Wang, L. Wu, F. Song, J. Zhou, *Phys. Chem. Chem. Phys.*, ****2023****, *25*, 12231–12244.

[13] K. Zhang, Y. Liu, J. Deng, S. Xie, X. Zhao, J. Yang, Z. Han, H. Dai, *Appl. Catal. B: Environ.*, ****2018****, *224*, 350–359.

[14] R. Guo, Y. Wang, J. Li, X. Cheng, D. D. Dionysiou, *Appl. Catal. B: Environ.*, ****2020****, *278*, 119297.

[15] Z. Xie, G. Xiao, X. Zeng, M. Yang, J. Yao, *Sep. Purif. Technol.*, ****2023****, *315*, 123481.

[16] P. Qiu, Z. Cheng, N. Xue, Y. Zeng, X. Kai, S. Zhang, C. Xu, F. Liu, Z. Guo, *Carbon*, ****2021****, *178*, 81–91.

[17] Q. Hou, M. Wang, T. Li, Y. Hou, K. Xuan, Y. Hao, *Chem. Eng. J.*, ****2023****, *464*, 142566.

[18] C. Liu, H. Dai, C. Tan, Q. Pan, F. Hu, X. Peng, *Appl. Catal. B: Environ.*, ****2022****, *310*, 121326.

[19] F. Liu, J. Cao, Z. Yang, W. Xiong, Z. Xu, P. Song, M. Jia, S. Sun, Y. Zhang, X. Zhong, *J. Colloid Interf. Sci.*, ****2021****, *581*, 195–204.

[20] S. Xin, G. Liu, X. Ma, J. Gong, B. Ma, Q. Yan, Q. Chen, D. Ma, G. Zhang, M. Gao, *Appl. Catal. B: Environ.*, ****2021****, *280*, 119386.

[21] C. Liu, H. Dai, C. Tan, Q. Pan, F. Hu, X. Peng, *Appl. Catal. B: Environ.*, ****2022****, *310*, 121326.

[22] Z. Zhang, J. Wang, H. Zhang, Y. Deng, B. Tan, J. Wang, T. Yang, W. Wang, *Appl. Surf. Sci.*, ****2024****, *671*, 160717.

[23] C. Ding, Y. Lu, J. Guo, W. Gan, S. Qi, Z. Yin, M. Zhang, Z. Sun, *Chem. Eng. J.*, ****2022****, *450*, 138271.

[24] N. Wang, X. Zhai, Y. Zhang, Q. Jiang, F. Ma, N. Liu, P. Ju, J. Duan, B. Hou, *J. Phys. Chem. Solids.*, ****2024****, *189*, 111977.

[25] X. Chen, C. Peng, F. Luo, G. Du, Y. Zhang, J. Zhao, L. Jiang, H. Su, S. Shan, T. Hu, *J. Alloy. Compd.*, ****2024****, *976*, 173369.

[26] X. Li, Y. Guo, L. Yan, T. Yan, W. Song, R. Feng, Y. Zhao, *Chem. Eng. J.*, ****2022****, *429*, 132234.

[27] F. Nie, W. Xu, D. Zhang, J. Wang, R. Zhang, X. Fang, Y. Wang, *J. Environ. Chem. Eng.*, ****2022****, *10*, 107604.

[28] C. Wang, Y. Li, Y. Wang, Y. Zhang, J. Feng, X. An, R. Wang, Y. Xu, X. Cheng, *J. Hazard. Mater.*, ****2024****, *466*, 133535.

[29] L. Wu, Y. Jiang, Z. Peng, X. Wang, C. Hou, Y. Liu, K. Chen, *J. Environ. Chem. Eng.*, ****2024****, *12*, 2024.

[30] X. Song, J. Tian, J. Ma, J. Ni, D. Liu, W. Wang, W. Shi, Y. Yuan, F. Cui, Z. Chen, *Chem. Eng. J.*, ****2023****, *453*, 139830.

[31] M. Xu, J. Deng, A. Cai, X. Ma, J. Li, Q. Li, X. Li, *Chem. Eng. J.* ****2020****, *384*, 123320.

[32] Z. Luo, Y. Yan, R. Spinney, D. D. Dionysiou, F. A. Villamena, R. Xiao, D. Vione, *Water Res.* ****2024****, *261*, 122023.

[33] J. Ma, H. Zhou, S. Yan, W. Song, *Water Res.* ****2019****, *149*, 56–64.
